# Transient Glycolytic Complexation of Arsenate Enhances Resistance in the Enteropathogen Vibrio cholerae

**DOI:** 10.1128/mbio.01654-22

**Published:** 2022-09-14

**Authors:** Emilio Bueno, Víctor Pinedo, Dhananjay D. Shinde, André Mateus, Athanasios Typas, Mikhail M. Savitski, Vinai C. Thomas, Felipe Cava

**Affiliations:** a Laboratory for Molecular Infection Medicine Sweden, Department of Molecular Biology, Umeå Centre for Microbial Research, Umeå University, Umeå, Sweden; b Center for Staphylococcal Research, Department of Pathology and Microbiology, University of Nebraska Medical Center, Omaha, Nebraska, USA; c Genome Biology Unit, European Molecular Biology Laboratory (EMBL), Heidelberg, Germany; University of Glasgow; University of Hawaii at Manoa

**Keywords:** arsenate, arsenite, enteric pathogens, Transposon-seq, *Vibrio cholerae*

## Abstract

The ubiquitous presence of toxic arsenate (As^V^) in the environment has raised mechanisms of resistance in all living organisms. Generally, bacterial detoxification of As^V^ relies on its reduction to arsenite (As^III^) by ArsC, followed by the export of As^III^ by ArsB. However, how pathogenic species resist this metalloid remains largely unknown. Here, we found that Vibrio cholerae, the etiologic agent of the diarrheal disease cholera, outcompetes other enteropathogens when grown on millimolar concentrations of As^V^. To do so, V. cholerae uses, instead of ArsCB, the As^V^-inducible *vc1068-1071* operon (renamed *var* for vibrio arsenate resistance), which encodes the arsenate repressor ArsR, an alternative glyceraldehyde-3-phosphate dehydrogenase, a putative phosphatase, and the As^V^ transporter ArsJ. In addition to Var, V. cholerae induces oxidative stress-related systems to counter reactive oxygen species (ROS) production caused by intracellular As^V^. Characterization of the *var* mutants suggested that these proteins function independently from one another and play critical roles in preventing deleterious effects on the cell membrane potential and growth derived from the accumulation As^V^. Mechanistically, we demonstrate that V. cholerae complexes As^V^ with the glycolytic intermediate 3-phosphoglycerate into 1-arseno-3-phosphoglycerate (1As3PG). We further show that 1As3PG is not transported outside the cell; instead, it is subsequently dissociated to enable extrusion of free As^V^ through ArsJ. Collectively, we propose the formation of 1As3PG as a transient metabolic storage of As^V^ to curb the noxious effect of free As^V^. This study advances our understanding of As^V^ resistance in bacteria and underscores new points of vulnerability that might be an attractive target for antimicrobial interventions.

## INTRODUCTION

Arsenic is a toxic metalloid commonly found in aquatic and terrestrial environments (1–3) as arsenate (As^V^) or its reduced form, arsenite (As^III^). The potent toxicity of this element makes it one of the best-studied natural poisons that impact public health (4). The mechanism of As^V^ toxicity is due to its structural resemblance to phosphate oxyanions and its uptake into the cell through phosphate transport channels (5, 6). Once in the cytoplasm, As^V^ can replace phosphate in energy-generating reactions, resulting in the formation of ADP-As^V^ instead of ATP by the ATP synthase and through substrate-level phosphorylation during glycolysis (7, 8). Conversely, cellular toxicity by As^III^ is exerted by a different mechanism. As^III^ can interact directly with thiol groups of proteins and other molecules, thereby interfering with diverse cellular processes (9, 10). Despite its risk to public health, arsenic has been used as an antimicrobial agent to treat infectious diseases (1, 4, 11, 12), increasing the selective pressure for microbes to acquire arsenic resistance.

Bacterial strategies to resist As^V^ include chelation by metal-binding metallothioneins (13), methylation to less toxic and more volatile arsenic derivatives (14), respiration (15), and extrusion of As^V^ or As^III^ by specific efflux pumps (e.g., ArsJ and ArsB, respectively) (9, 16–18). In bacteria, the most common mechanism of As^V^ resistance genes occurs via As^V^ reduction to As^III^, followed by the extrusion of the latter through an As^III^ transporter (19). As^V^ resistance genes are commonly organized in operons, e.g., the *arsRDABC* operon from Escherichia coli R773 (19). In this example, *arsC* encodes a dedicated arsenate reductase, and *arsB* encodes an efflux pump of As^III^ (20). The *arsR* gene encodes the repressor of the *ars* operon. When As^III^ is present, its interaction with ArsR releases it from the *ars* promoter, thus increasing transcription of the operon. *arsA* encodes an ATPase that, together with ArsB, forms the As^III^ extrusion system. Alternatively, As^III^ extrusion via ArsB can be driven by proton motive force in bacterial species lacking ArsA. Finally, *arsD* encodes a chaperone that enhances arsenic extrusion by transferring As^III^ to ArsAB (9).

Despite being extensively studied in environmental soil and marine bacterial species, the mechanisms underlying As^V^ resistance in human-pathogenic bacteria remain largely unknown. Here, we investigated As^V^ resistance in representative enteric pathogens and found that Vibrio cholerae exhibits remarkably high resistance to this metalloid. Using genome mining and functional characterization, we demonstrated that V. cholerae does not detoxify As^V^ by reducing it to As^III^. Instead, transposon-based functional screens identified the operon *vc1068-1071* as the primary genetic determinant of As^V^ resistance in V. cholerae. Mechanistic characterization of this system revealed that resistance to As^V^ in V. cholerae is mediated by a transient metabolic complexation of As^V^ with the glycolytic intermediate 3-phosphoglycerate (3PG) to generate 1-arseno-3-phosphoglycerate (1As3PG). Our results support that this As^V^-containing metabolite is transient in the cell: its formation cushions the cellular damage caused by free As^V^, while its dissociation enables V. cholerae to release free As^V^ through the ArsJ efflux permease.

## RESULTS

### High resistance to arsenate in V. cholerae is independent of the ArsC reductase.

**FIG 1:**
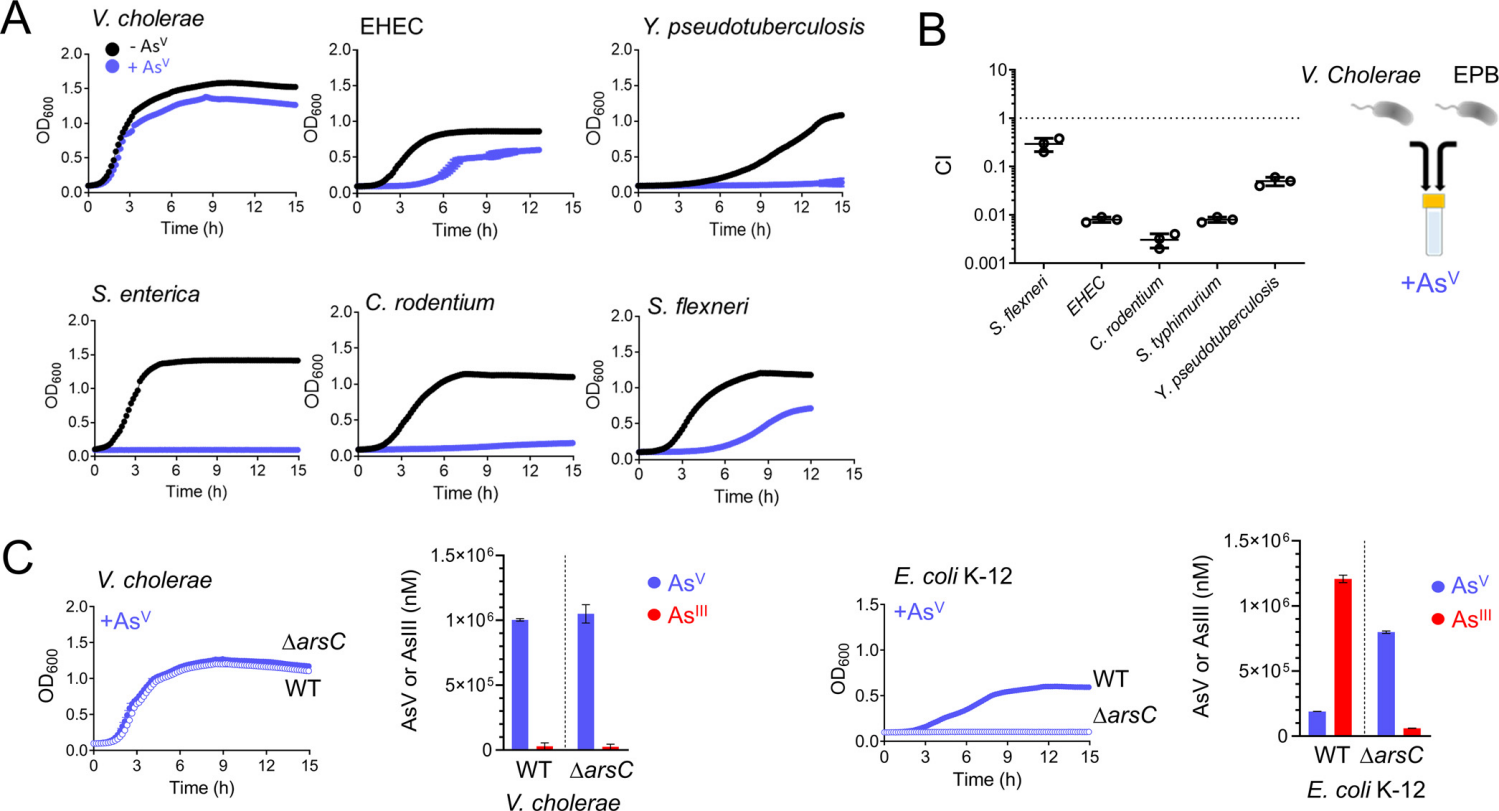
V. cholerae is the enteric pathogen with the greatest resistance to As^V^. (A) Growth curves (OD_600_) of the enteric pathogens V. cholerae, enterohemorrhagic Escherichia coli (EHEC), Salmonella enterica, Citrobacter rodentium, Shigella flexneri, and Yersinia pseudotuberculosis. Cultures were grown in LB medium in the absence (black) and in the presence (blue) of 10 mM arsenate (As^V^). (B) *In vitro* competition among V. cholerae and other enteropathogenic bacteria (EPB) studied in panel A. Cells were incubated in LB medium supplemented with 10 mM As^V^ for 8 h. CI values from competition in the presence of As^V^ were normalized with respect to CI values in the absence As^V^. (C) Growth curves (OD_600_) of WT (blue filled circles) and *arsC* (blue empty circles) mutant strains from V. cholerae and E. coli K-12. Cultures were grown in LB medium supplemented with 10 mM As^V^. As^V^ and As^III^ concentrations were determined by ICP-MS from supernatants of V. cholerae and E. coli WT and *arsC* mutant strains after 8 h of incubation in 1.5 mM As^V^. Data are the mean of three biological replicates ± the standard error of the mean (SEM).

While studying the tolerance of enteropathogenic bacteria to metalloids, we observed that Vibrio cholerae, the causative agent of cholera, was able to grow in media supplemented with supraphysiological (30 mM) concentrations of arsenate (As^V^) (see Fig. S1 In the supplemental material). This level of resistance was unmatched by other human enteric pathogens, as lower concentrations of As^V^ (10 mM) readily caused total or partial growth inhibition of Salmonella enterica, Citrobacter rodentium, Yersinia pseudotuberculosis, enterohemorrhagic E. coli (EHEC), and Shigella flexneri (Fig. 1A). Consistently, V. cholerae outcompeted these species by 5- to 500-fold in coculture experiments grown in media supplemented with As^V^ (Fig. 1B).

10.1128/mbio.01654-22.1 FIG S1 Growth curves (OD_600_) of V. cholerae WT in the presence of As^V^. V. cholerae WT was grown in LB medium in the presence of 10, 20, and 30 mM As^V^. Data are the mean of three biological replicates ± SEM. Download FIG S1, TIF file, 0.1 MB. Copyright © 2022 Bueno et al. 2022 Bueno et al. https://creativecommons.org/licenses/by/4.0/ This content is distributed under the terms of the Creative Commons Attribution 4.0 International license.

To investigate the molecular mechanisms of As^V^ resistance in V. cholerae, we first searched for canonical determinants for As^V^ detoxification in its genome. V. cholerae *vc2165* encodes a homolog of E. coli K-12 arsenate reductase ArsC, including the conserved catalytic cysteine and arginine residues (21, 22) (Fig. S2A). However, while E. coli’s ArsC enzyme is critical for resistance to As^V^, inactivation of *vc2165* did not compromise V. cholerae growth in the presence of this metalloid (Fig. 1C). Thus, we reasoned that V. cholerae’s ArsC might not be active or expressed. To measure ArsC activity, we quantified extracellular As^V^ and As^III^ by inductively coupled plasma mass spectrometry (ICP-MS) and observed that while E. coli reduced ~80% of As^V^ to As^III^ in an ArsC-dependent manner, V. cholerae produced no As^III^ (Fig. 1C). Interestingly, replacement of the V. cholerae *arsC* allele by that of E. coli caused growth inhibition, likely due to toxic As^III^ formation, as growth was recovered by expressing E. coli’s As^III^-transporter, ArsB (Fig. S2B). Altogether, these results indicate that V. cholerae ArsC is inactive and that As^V^ resistance in this bacterium is independent of the ArsC-ArsB system that relies on the production and elimination of As^III^.

10.1128/mbio.01654-22.2 FIG S2 Characterization of V. cholerae ArsC. (A) Schematic of the *arsC*-containing operons from E. coli K-12 and V. cholerae and protein sequence alignments. Black arrows depict ArsC catalytic cysteine and arginine residues implicated in As^V^ reduction to As^III^. (B) Growth curves (OD_600_) of V. cholerae WT and two derivative strains where *arsC* has been replaced by E. coli’s *arsC* (red, *arsC*^ec^) and *arsC*^ec^ strain where *arsB* is overexpressed in *trans* (blue, *arsC^ec^ + arsB^ec^*) (see bottom panel and Materials and Methods for further details). Cultures were grown in LB medium in the presence 10 mM As^V^. (Bottom) Explanatory diagrams illustrating the expected output from each construct. Data are the mean of three biological replicates ± SEM. Download FIG S2, TIF file, 2.3 MB. Copyright © 2022 Bueno et al. 2022 Bueno et al. https://creativecommons.org/licenses/by/4.0/ This content is distributed under the terms of the Creative Commons Attribution 4.0 International license.

### Resistance to As^V^ in V. cholerae depends on the *vc1068*-*vc1071* operon.

**FIG 2:**
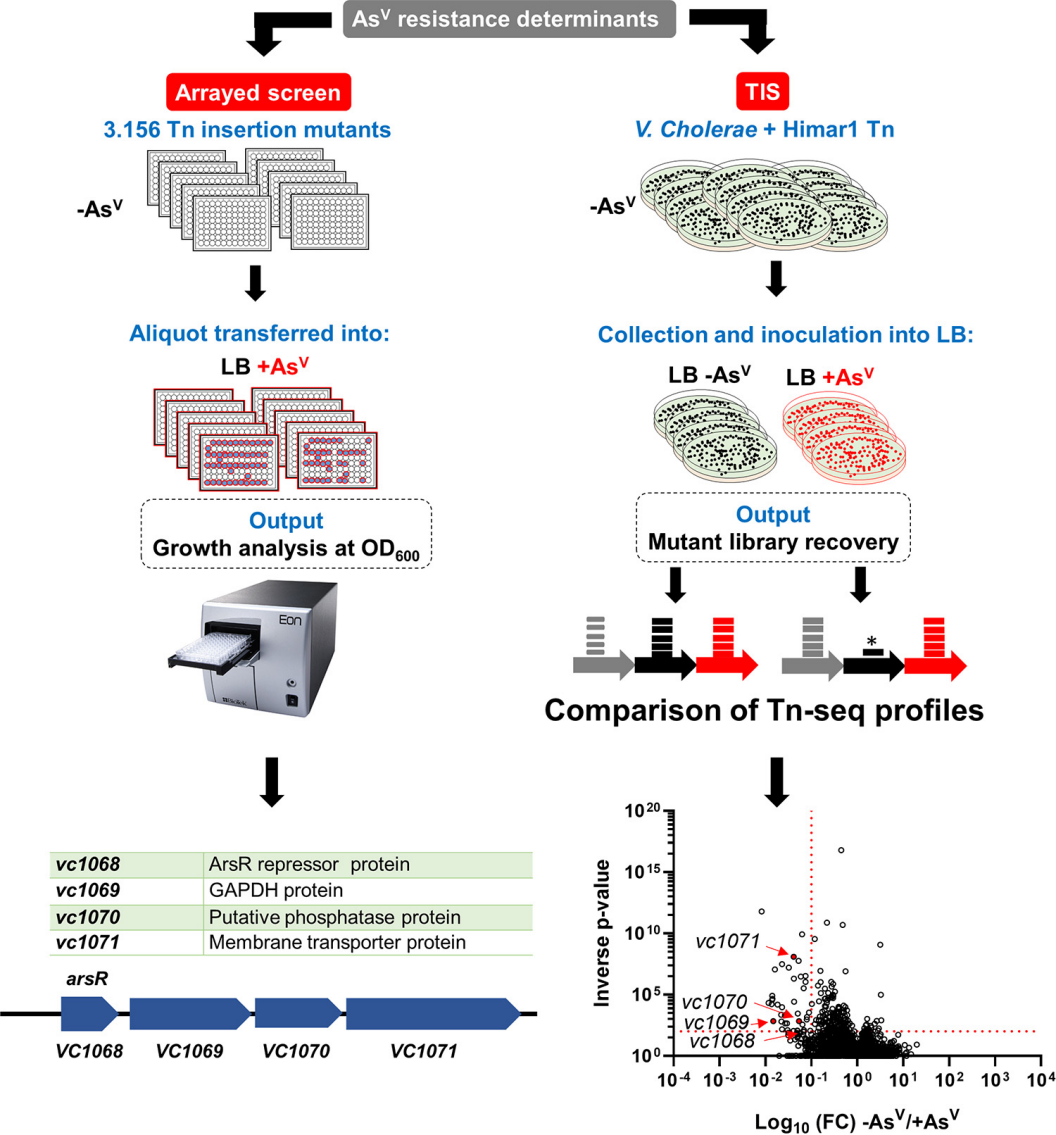
Identification of essential genetic determinants for As^V^ resistance in V. cholerae. (Left) Experimental workflow for an arrayed transposon mutant screen to identify the genes required for As^V^ resistance in V. cholerae and the list of mutant strains identified. (Right) Experimental workflow for TIS screen in the presence and absence of 1 mM As^V^. Volcano plot depicting the ratio of read counts mapped to individual genes in transposon libraries of (V. cholerae plated onto medium supplemented with As^V^). Red dotted lines indicate arbitrary thresholds of a fold change of (FC) <0.1 and an inverse *P* value of >20. Genes in red show loci also identified in the arrayed screen (see Table S1 for an extended version).

To identify the genetic determinants conferring resistance to As^V^ in V. cholerae, we performed two complementary genome-wide screenings based on (i) transposon insertion sequencing (TIS) and (ii) the use of a previously described V. cholerae transposon mutant library (23) (Fig. 2). Both screens permit assessment of the mutants’ relative fitness. While screening of the V. cholerae transposon mutant library assesses fitness of individual mutants in separated wells, TIS valuates subtle fitness differences during growth under competition with other mutants from the Tn-library. In TIS, a V. cholerae transposon library containing ~500,000 mutants is generated and incubated in parallel with and without AsV (see representation in Fig. 2). Transposon Q absent after incubation with AsV indicate the essentiality of those determinants to survive under such condition.

10.1128/mbio.01654-22.8 TABLE S1 Complete list of genes identified as underrepresented by TIS in the presence of 1 mM As^V^ with thresholds of a fold change of <0.1 and an inverse *P* value of s>75. Download Table S1, DOCX file, 0.01 MB. Copyright © 2022 Bueno et al. 2022 Bueno et al. https://creativecommons.org/licenses/by/4.0/ This content is distributed under the terms of the Creative Commons Attribution 4.0 International license.

Only four nonessential Transposon mutants (*vc1068*::*tn*, *vc1069*::*tn*, *vc1070*::*tn*, and *vc1071*::*tn*) were mutually underrepresented in both screenings (Fig. 2). We renamed *vc1068-71 var* for *vibrio* arsenate resistance cluster for reasons described below. *vc1068* encodes a homolog of the ArsR repressor, *vc1069* (renamed *varG*) encodes a putative glyceraldehyde-3-phosphate dehydrogenase (GAPDH), *vc1070* (renamed *varH*) encodes an uncharacterized putative phosphatase, and *vc1071* encodes an ArsJ-like As^V^ transporter (17). As expected, the *arsC* mutant was not identified in the screens, confirming that V. cholerae does not detoxify As^V^ by reducing it to As^III^. Although homologues to VarG and ArsJ were previously reported in Pseudomonas aeruginosa, their function in As^V^ resistance has not been studied *in vivo* (17).

**FIG 3:**
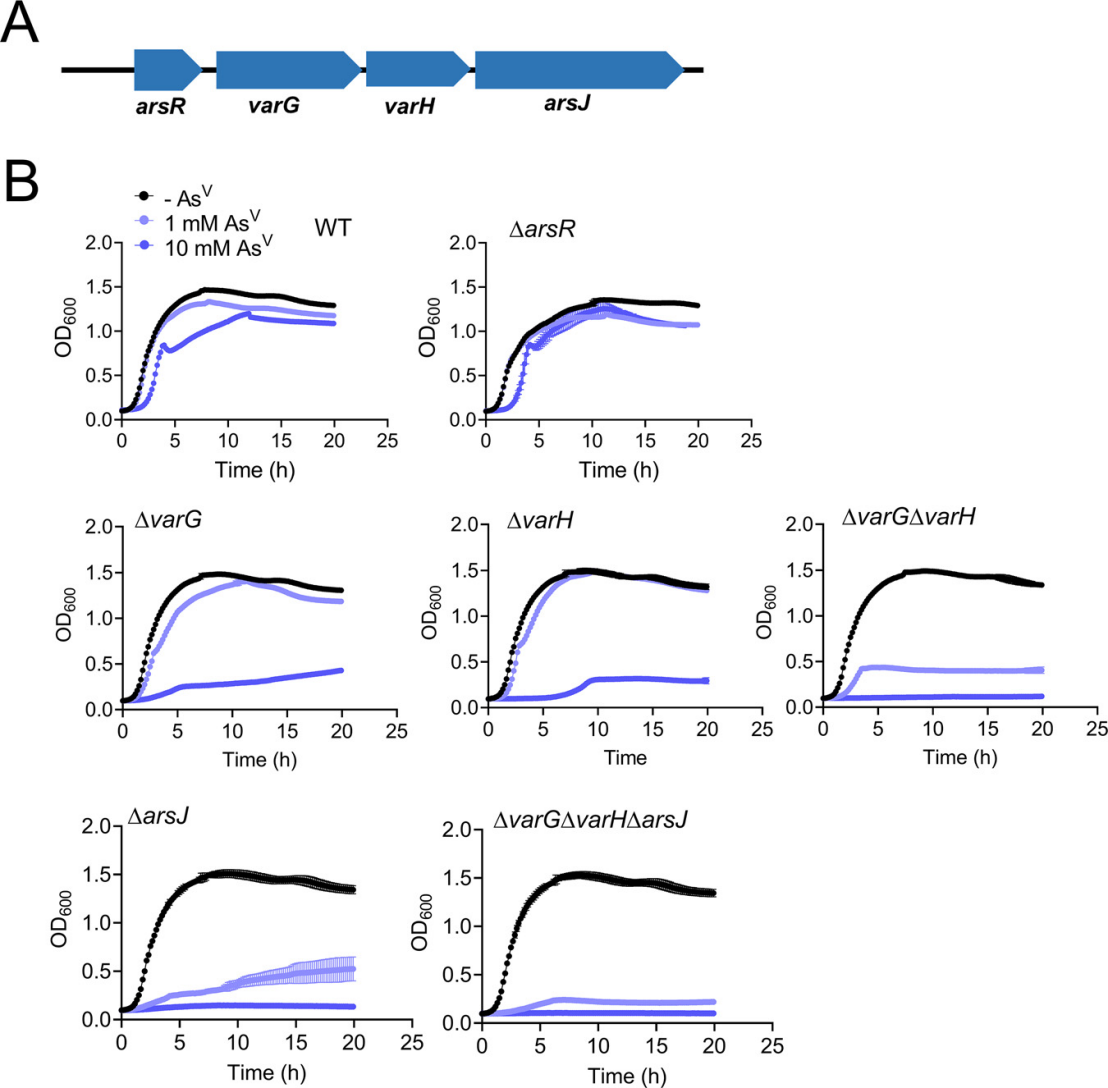
Phenotypic characterization of the V. cholerae *var* operon. (A) Var operon. (B) Growth curves (OD_600_) of V. cholerae WT, single, and combinatorial *var* mutant strains. Cultures were grown in LB medium in the absence or presence of 1 and 10 mM As^V^. Data are the mean of three biological replicates ± SEM.

To verify the implication of the *var* cluster on As^V^ resistance, we constructed individual in-frame markerless deletion mutants. All mutants were sensitive to As^V^ except for Δ*arsR* (Fig. 3A), suggesting that the conditional lethality of the *arsR*::tn was likely due to the polar effect on the expression of the downstream genes *varG*, *varH*, and *arsJ*. Indeed, transcriptional analysis using fusions of the *lacZ* reporter to potential promoter regions located upstream of each *var* gene demonstrated that expression of this cluster depends on As^V^, and it is driven from a single promoter upstream of *arsR* (Fig. S3). Inactivation of *arsR* turned transcription from this promoter constitutive (in both the presence and absence of As^V^), confirming that VC1068 is the V. cholerae ArsR repressor of the *var* operon (Fig. S3). Further characterization revealed that Δ*arsJ* presents a more dramatic growth defect than the rest of the *var* mutants at low As^V^ concentrations (1 mM) (Fig. 3). However, at 10 mM As^V^, the growth of the *varG* and *varH* mutant strains was also compromised despite the presence of ArsJ. These results suggest that the ArsJ transporter is the main determinant for As^V^ resistance in V. cholerae, but VarG and VarH also become relevant at higher concentrations of As^V^. Furthermore, the inactivation of *varG*, *varH*, and *arsJ* (double and triple mutants) aggravated growth defects exhibited by the single mutants, suggesting complementary/synergistic functions for these proteins in As^V^ resistance in V. cholerae (Fig. 3A).

10.1128/mbio.01654-22.3 FIG S3 Transcriptional analysis of the *var* operon. β-galactosidase activity from V. cholerae WT and *arsR* mutant (Δ*arsR*) strains carrying DNA regions upstream of *arsR, varG*, *varH*, and *arsJ* genes fused to the *lacZ* reporter gene. Each DNA region fused to *lacZ* is depicted by arrows. Cultures were grown in LB medium in the presence or absence of 10 mM As^V^. Data are the mean of three biological replicates ± SEM. Download FIG S3, TIF file, 0.2 MB. Copyright © 2022 Bueno et al. 2022 Bueno et al. https://creativecommons.org/licenses/by/4.0/ This content is distributed under the terms of the Creative Commons Attribution 4.0 International license.

### VarG is an As^V^-inducible GAPDH that preferentially binds As^V^.

**FIG 4:**
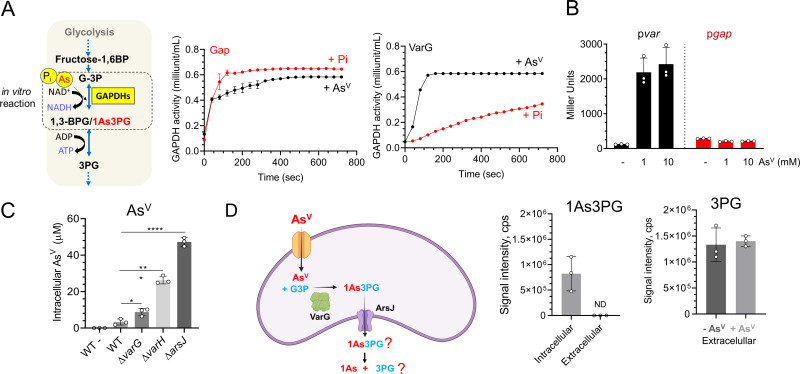
Characterization of V. cholerae VarG. (A) *In vitro* GAPDH activity (OD_450_) of purified V. cholerae VarG and the glycolytic Gap enzymes using P_i_ or As^V^ as the substrate. (B) β-galactosidase activity of strains in which the *lacZ* reporter gene is cloned under the control of the *var* operon and the *gap* gene promoters. Cultures were grown in LB medium in the absence or presence of 1 and 10 mM As^V^ for 5 h. (C) Intracellular As^V^ concentrations of WT, *varG*, *varH*, and *arsJ* mutant strains grown with 1 mM As^V^ by ICP-MS. (D) (Left) As^V^ detoxification model in P. aeruginosa DK2 (1). (Right) Quantification of 1As3PG and 3PG from intracellular and extracellular samples of V. cholerae cells grown with and without 1 mM As^V^. Data are the mean of three biological replicates ± SEM.

V. cholerae VarG presents a high degree of protein sequence (E value, 5^e-86^) (Fig. S4A) and structure (AlphaFold2 TM-score, 0.96) (Fig. S4B), similar to the glycolytic GAPDH VC2000 (Gap), including the conserved catalytic Cys residue (24). To characterize VarG’s activity compared to the glycolytic paralogue Gap in particular, we purified both proteins from V. cholerae and performed *in vitro* GAPDH activity assays (25) in the presence or absence of As^V^. While both proteins exhibited GAPDH activity, VarG showed preference for As^V^ over P_i_ (the canonical substrate) (Fig. 4A). Conversely, the glycolytic GAPDH Gap performed better with P_i_. Furthermore, while the expression of *Gap* remained low and constitutive, transcription from the *var* promoter was strongly induced by As^V^ (Fig. 4B). Therefore, these results suggest that while V. cholerae Gap is active on As^V^, this enzyme-specific activity and the protein levels are likely insufficient to replace VarG essentiality in V. cholerae resistance to As^V^.

10.1128/mbio.01654-22.4 FIG S4 V. cholerae VarG presents a high degree of similarity to the glycolytic GAPDH Gap. (A) Sequence alignments of the V. cholerae VarG and the glycolytic GAPDH proteins using Vector NTI software. (B) (Left) Predicted structures of VarG using AlphaFold2 (https://colab.research.google.com/github/sokrypton/ColabFold/blob/main/AlphaFold2.ipynb). The region of the protein structure depicted in blue indicates the least error in prediction. (Right) VarG and Gap structures overlapping using TM-score (https://zhanggroup.org/TM-score/). A TM-score value of 1 indicates 100% structural identity among proteins. Download FIG S4, TIF file, 2.7 MB. Copyright © 2022 Bueno et al. 2022 Bueno et al. https://creativecommons.org/licenses/by/4.0/ This content is distributed under the terms of the Creative Commons Attribution 4.0 International license.

### Arsenate detoxification in V. cholerae is facilitated by extrusion of free arsenate instead of being complexed as 1-arseno-3-phosphoglycerate.

A VarG homolog from P. aeruginosa was proposed to function as a GAPDH that complexes As^V^ with glyceraldehyde-3-phosphate (G3P) into 1-arseno-3-phosphoglycerate (1As3PG), which is further eliminated through ArsJ (17). However, these experiments were performed *in vitro* using a commercial glycolytic GAPDH from rabbit instead of the As^V^-specific GAPDH from P. aeruginosa DK2. If this model applies to V. cholerae, the absence of either VarG or ArsJ will similarly affect AsV export and resistance. However, our data show that Δ*varG* did not phenocopy the growth defect of Δ*arsJ* (Fig. 3). Furthermore, analysis of intracellular levels of As^V^ by ICP-MS revealed that, in relation to the wild type (WT), while the Δ*varG* strain only showed moderate (ca. 3×) accumulation of As^V^, inactivation of ArsJ accumulated this metalloid ca. 20× (Fig. 4C). Although these results suggest that VarG is not essential to export As^V^, we cannot dismiss the possibility of As^V^ being complexed and extruded as 1As3PG in V. cholerae.

Using high-resolution mass spectrometry (HRMS), we detected a mass (*m/z*) of 304.8696 Da, specific to the cultures supplemented with As^V^, which was compatible with 1As3PG (*m/z* 304.8674, *m/z *< 0.003 Da difference between experimental and theoretical mass) (Fig. 4D and Fig. S5). 1As3PG was also detected in Δ*varG*, consistent with the ability of the constitutively expressed glycolytic Gap GAPDH to use As^V^ (Fig. S5). 1As3PG was detected in intracellular cytoplasmic fractions but not in the extracellular milieu, suggesting that this molecule is not expelled in V. cholerae (Fig. 4D and Fig. S5). As it has been reported that 1As3PG is rapidly dissociated to As^V^ and 3PG (26), we reasoned that if exported, its decomposition should increase extracellular 3PG levels in relation to cultures without As^V^. However, we found no significant differences in the levels of extracellular 3PG between conditions, supporting the idea that As^V^ detoxification likely occurs through the release of free As^V^ rather than 1As3PG through ArsJ (Fig. 4C and Fig. S5).

10.1128/mbio.01654-22.5 FIG S5 Quantification of intra- and extracellular metabolites from V. cholerae. Representative plots from identification of 1As3PG and 3PG from intracellular and extracellular samples from V. cholerae WT and Δ*varG* cells were grown with and without 1 mM As^V^. The compounds were separated by UPLC using an XSELECT HSS XP column. 1As3PG was identified using a Thermo Orbitrap Exploris 480 high-resolution mass spectrometer, and 3PG was identified using a triple-quadrupole-ion trap hybrid mass spectrometer QTRAP 6500+ (SCIEX, USA) connected with a Waters ultraperformance liquid chromatography I-class (UPLC) system. Download FIG S5, TIF file, 1.1 MB. Copyright © 2022 Bueno et al. 2022 Bueno et al. https://creativecommons.org/licenses/by/4.0/ This content is distributed under the terms of the Creative Commons Attribution 4.0 International license.

### VarH is involved in 1As3PG dissociation and arsenate export.

**FIG 5:**
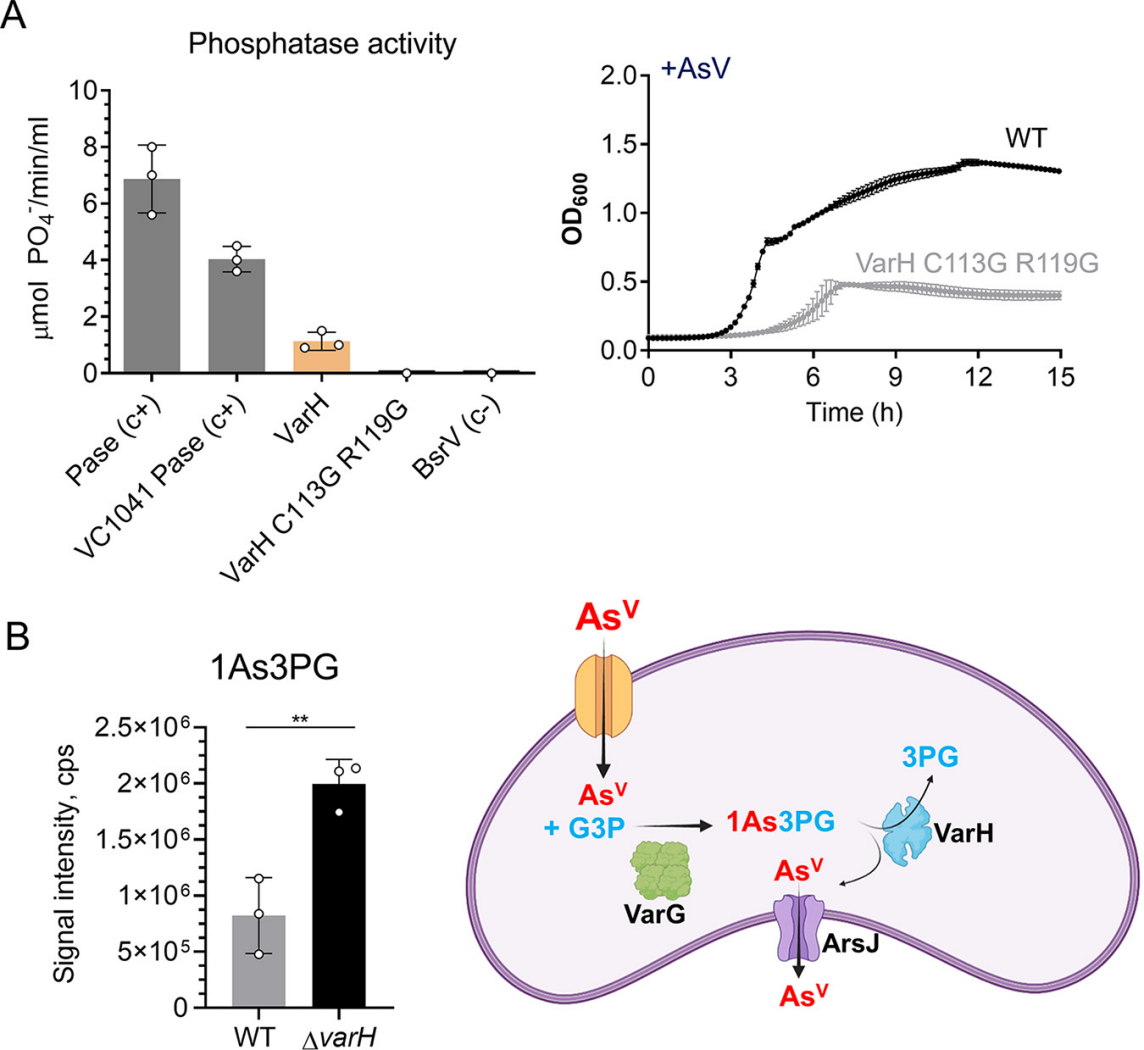
Characterization of V. cholerae VarH. (A) (Left) *In vitro* phosphatase activity of purified V. cholerae VarH and catalytic mutant derivative where cysteine 113 and arginine 119 were replaced by glycine. V. cholerae protein-tyrosine-phosphatase (VC1041) and broad-spectrum racemase (BsrV) were used as positive and negative controls, respectively. (Right) Growth curves (OD_600_) of V. cholerae WT and a VarH catalytic mutant derivative. Cultures were grown in LB medium in the presence of As^V^. (B) (Left) Intracellular 1As3PG concentrations from V. cholerae WT and Δ*varH* cells grown with and without 1 mM As^V^ by HRMS. (Right) Schematic of putative mechanism for As^V^ detoxification in V. cholerae. Data are the mean of three biological replicates ± SEM.

To find clues that help us understand the role that 1As3PG plays in V. cholerae’s resistance to As^V^, we investigated VarH. Structural *in silico* analysis using the phyre2 tool (www.sbg.bio.ic.ac.uk/phyre2) and AlphaFold2 revealed that VarH is a cysteine-based protein tyrosine phosphatase (PTP) belonging to the dual-specificity phosphatase superfamily that presents a very high topology similarity to the human Kap PTP phosphatase (Fig. S6A and B). To determine if VarH has phosphatase activity, we purified this protein and assessed its capacity to dephosphorylate the chromogenic substrate pNPP. Our results show that, compared to the wheat phosphatase or V. cholerae’s PTP VC1041 positive controls, VarH presents lower phosphatase activity, which turned off upon replacement of the putative catalytic cysteine and arginine by glycine residues (Fig. 5A, left panel). Furthermore, consistent with the importance of these residues in VarH activity, replacement of the WT *varH* allele by the catalytically inactive variant *varH* C113G/R119G impaired V. cholerae growth in the presence of As^V^ (Fig. 5A, right panel). These results suggest that the phosphatase activity of VarH is required to provide As^V^ resistance in V. cholerae.

10.1128/mbio.01654-22.6 FIG S6 VarH characterization. (A) Sequence alignment of the secondary structure of the VarH protein with the crystal structure of the human Kap PTP phosphatase using the software Phyre2 (www.sbg.bio.ic.ac.uk/phyre2). The human Kap structure matched VarH with 100% confidence and 99% coverage. Amino acid sequences within the black box (from the amino acid 113 [H] onwards) indicate the conserved catalytic loop [C(x)_5_R] of protein tyrosine phosphatase PTP. Green and black coloured boxes indicate deletions and insertions, respectively, with respect to the template sequence. (B) Predicted structures of VarH with AlphaFold2 (https://colab.research.google.com/github/sokrypton/ColabFold/blob/main/AlphaFold2.ipynb). The regions of the protein structure depicted in blue indicate no error in prediction. (Right) Overlap between VarH protein structure obtained with AlphaFold2 and crystal structure of the Kap PTP protein using TM-score (https://zhanggroup.org/TM-score/). A TM-score value of 1 represents 100% structural identity among proteins. (C) SDS-PAGE gel depicting the VarG His tag purified protein from the V. cholerae WT and *varH* mutant strains (see Materials and Methods for details). VarG proteins were excised from the gel, and its phosphorylation state was quantified by LC-MS/MS. (D) Phosphorylation state quantification of VarG from V. cholerae WT and *varH* mutant strains. S204, serine amino acid from VarG in position 204; Y108, tyrosine amino acid from VarG in position 108. (E) Representative plots from identification of 1As3PG and 1AsG from intracellular samples of V. cholerae WT and Δ*varH* cells grown with and without 1 mM As^V^. The compounds were separated by UPLC using an XSELECT HSS XP column. The detection of 1As3PG and 1AsG was performed using a Thermo Orbitrap Exploris 480 high-resolution mass spectrometer. Data are the mean of three biological replicates ± SEM. Download FIG S6, TIF file, 2.7 MB. Copyright © 2022 Bueno et al. 2022 Bueno et al. https://creativecommons.org/licenses/by/4.0/ This content is distributed under the terms of the Creative Commons Attribution 4.0 International license.

Since VarG and VarH are coregulated by As^V^ and both support resistance to this metalloid, we hypothesized that VarH could regulate VarG activity by modulating its phosphorylation state. To assess such a possibility, we purified VarG from V. cholerae WT and Δ*varH* (Fig. S6C) and analyzed VarG phosphorylation by liquid chromatography-tandem mass spectrometry (LC-MS/MS). However, even though VarG was phosphorylated on Tyr108 and Ser204, those phosphorylation levels were independent of VarH (Fig. S6D). Then, we reasoned that as P_i_ and As^V^ are analogs, VarH could potentially act on 1As3PG as a phosphatase to produce 1AsG + Pi or as an “arsenatase” to render 3PG + As^V^. Remarkably, we found that 1As3PG concentrations were significantly elevated in Δ*varH* compared to those in the WT strain (Fig. S6E), suggesting that VarH might use 1As3PG as a substrate. Although we could not confirm VarH’s activity *in vitro*, potentially due to the instability of 1As3PG (27), the fact that VarH is an active phosphatase but 1AsG is not detected in V. cholerae suggests that VarH could dissociate the 1As3PG complex into 3PG and free As^V^, which would be released through ArsJ.

### Intracellular arsenate impacts the V. cholerae proteome and leads to ROS accumulation and membrane potential defects.

**FIG 6:**
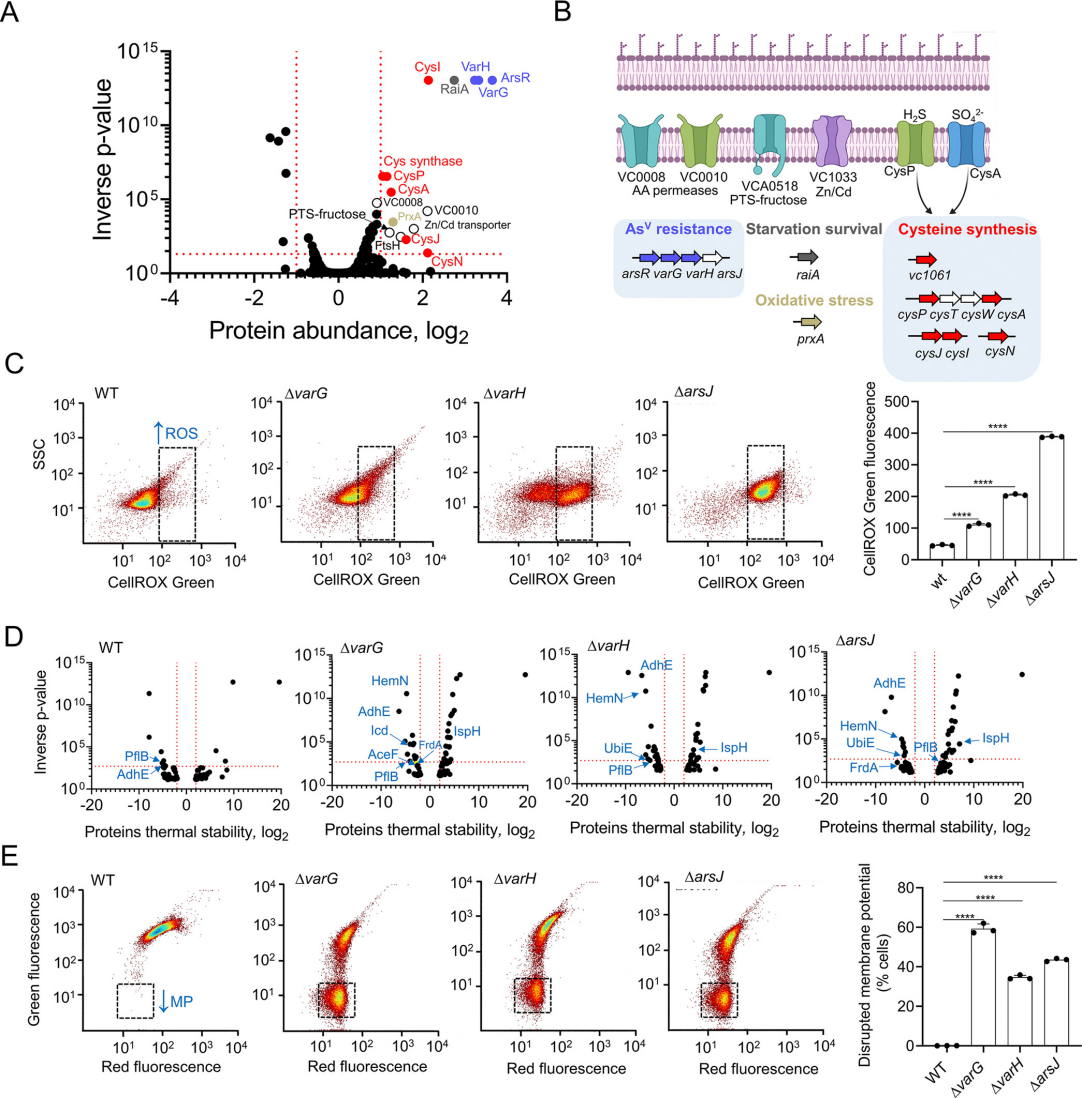
Oxidative stress response and membrane potential alterations of V. cholerae WT and the *var* mutants. (A) Volcano plot of LIMMA analysis output depicting read fold change (log_2_) of protein abundance and inverse *P* value for each protein queried in the proteomic screen in response to 1 mM As^V^ in V. cholerae WT. Red dotted lines indicate arbitrary thresholds of a fold change (log_2_) of >1 or <1 and an inverse *P* value of >20. Var proteins are labeled in blue and oxidative stress related proteins (cysteine biosynthesis and PrxA are labeled as in described in panel B). (B) Schematic of the systems induced in response to As^V^ in V. cholerae. White coloured genes depict undetected genes within an operon. (C) Representative dot plots from V. cholerae WT and *var* mutant cells stained with CellRox green following growth in the presence of 1 mM As^V^ for 5 h. The black dotted gate indicates an area with higher CellRox fluorescence (higher ROS levels). (D) Volcano plot of LIMMA analysis output depicting the read fold change (log_2_) of protein thermal stability and inverse *P* value for each protein queried in the proteomic screen in response to 1 mM As^V^ in V. cholerae WT and *var* mutant strains. As^V^ interacting proteins related to energy-generating pathways are labeled in blue. PflB, VC1866 formate acetyltransferase; AdhE, VC2033 alcohol dehydrogenase/acetaldehyde dehydrogenase; HemN, VC0116 oxygen-independent coproporphyrinogen III oxidase; Icd, VC1141 isocitrate dehydrogenase; FrdA, VC2656 fumarate reductase; IspH, 4-hydroxy-3-methylbut-2-enyl diphosphate reductase; AceF, VC2413 pyruvate dehydrogenase, E2 component; UbiE, VC0083 ubiquinone/menaquinone biosynthesis methyltransferase. (E) Representative dot plots from V. cholerae WT and *var* mutant cells stained with DiOC2 following growth in the presence of 1 mM As^V^ for 5 h. The black dotted gate indicates an area with lowered green fluorescence (lower membrane potential). Data are the mean of three biological replicates ± SEM.

To broadly assess global responses triggered by As^V^ in V. cholerae, we analyzed the pathogen proteome upon exposure to As^V^. Interestingly, a relatively low number of proteins (~20 proteins) were upregulated upon exposure to As^V^, indicating that adaptations to this metalloid might not require extensive proteome rewiring in V. cholerae. As expected, these proteins included the As^V^ resistance Var proteins but also amino acid permeases, a Zn/Cd transporter, members of the phosphotransferase system (PTS)-fructose system, the starvation stress response protein RaiA, and the cysteine synthesis and the peroxiredoxin PrxA (Fig. 6A and B). Cysteine is one of the main targets of reactive oxygen species (ROS) and a functional component of glutathione, which plays a crucial role in the adjustment of cellular redox potential (28). In this line, PrxA has been involved in the adaptation to hydrogen peroxide in V. cholerae (29). Therefore, we reasoned that the *var* mutants, which display increased As^V^ intracellular levels, might suffer from higher oxidative stress than the V. cholerae WT strain. To test this hypothesis, we measured ROS levels by analyzing fluorescence of a DNA dye (CellROX green) that emits fluorescence when it is oxidized by ROS in the V. cholerae WT and *var* mutant strains and observed a direct correlation between As^V^ accumulation and ROS level (Δ*arsJ* > Δ*varH* > Δ*varG* > WT) (Fig. 6C). These findings collectively indicate that AsV induces oxidative stress in V. cholerae, which is exacerbated in the *var* mutants due to a supraphysiological accumulation of this toxic metalloid.

In addition to ROS affecting Cys-containing proteins, we cannot dismiss a direct effect of As^V^ on the proteome of V. cholerae. To this end, we performed thermal proteome profiling (TPP), a method that globally analyzes multiple types of protein interactions (30). TPP data showed an increased thermal stability of multiple cytosolic proteins that could potentially interact with As^V^ (~3-fold) in the *var* mutants with respect to the WT (Fig. 6D and Table S1). As^V^-interacting protein candidates included proteins involved in the synthesis of heme groups of respiratory cytochromes, respiratory ubiquinone, fermentation, and carbon catabolism, suggesting that accumulation of As^V^ in Var-defective strains might interfere with cell bioenergetics. To investigate this possibility, we monitored the bacterial membrane potential in the presence of As^V^ using flow cytometry (Fig. 6E). Interestingly, inactivation of the *var* system resulted in a significant depolarization of the mutant’s membrane. Together, these results demonstrate that AsV can, directly and indirectly, affect the V. cholerae proteome, leading to oxidative stress and defects in cellular bioenergetics, both possibly related to the growth impairment of the *var* mutants.

### The V. cholerae *var* operon provides As^V^ resistance to enteric pathogens.

**FIG 7:**
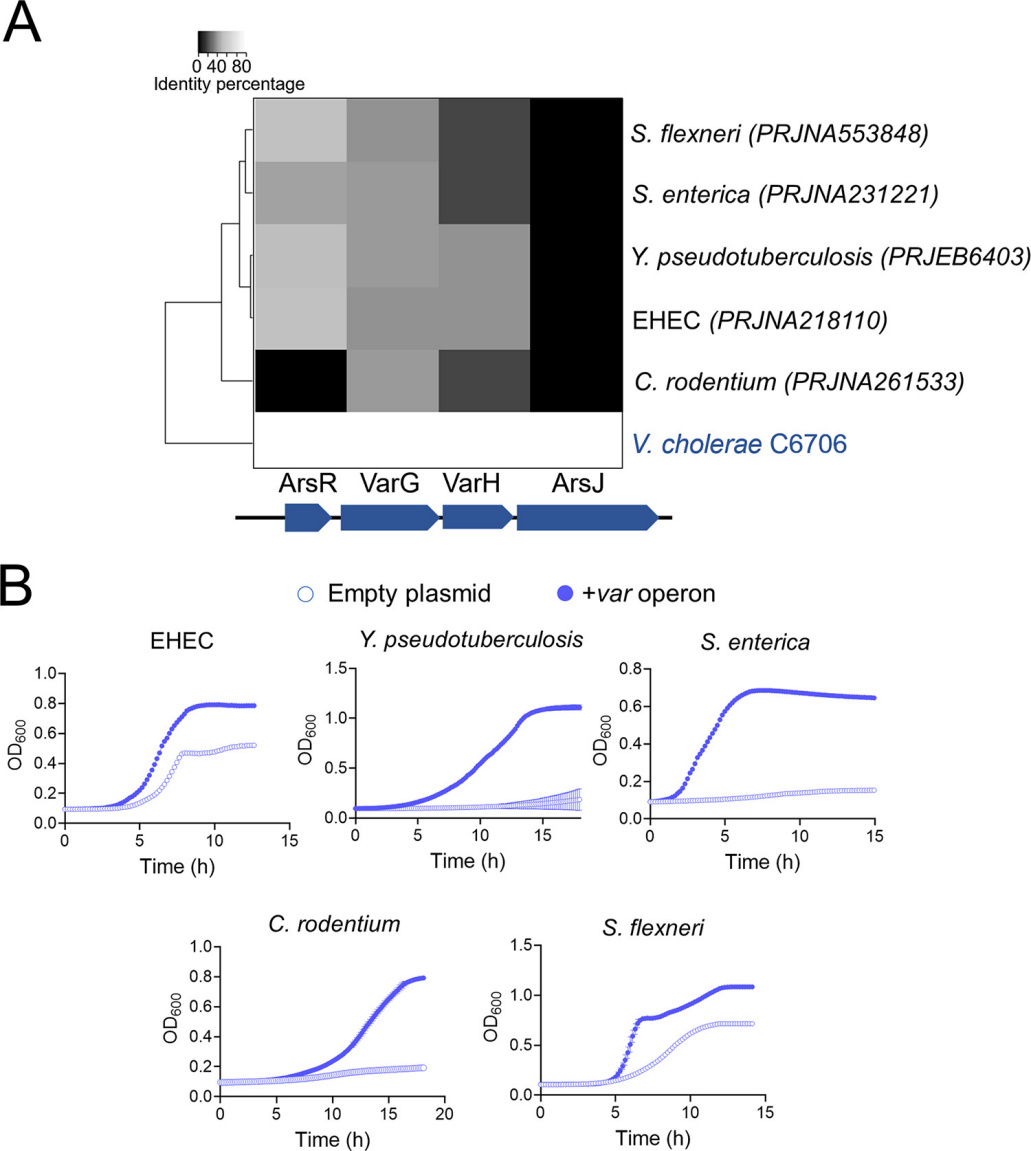
V. cholerae Var enhances As^V^ resistance in enteropathogenic bacteria. (A) Hierarchical heat map clustering of enteropathogenic bacteria with respect to V. cholerae based on the percentage identity of *var* genes. (B) Growth curves (OD_600_) of the As^V^-sensitive enteropathogenic bacteria clustered in panel A, carrying an inducible empty plasmid (empty circle) or the same plasmid expressing the V. cholerae *var* operon (blue filled circle). Cultures were grown in LB medium in the presence of 10 mM As^V^. Data from panel B are the mean of three biological replicates ± SEM.

*In silico* analysis showed that, except for C. rodentium, ArsR is the only As^V^ resistance determinant conserved between V. cholerae and the As^V^-sensitive enteric pathogens (Fig. 7A). In general, these enteropathogenic species detoxify As^V^ via ArsC-dependent reduction to As^III^ and subsequent extrusion through ArsB. As V. cholerae lacks the As^III^- production/extrusion tandem ArsCB, we wondered whether the *var* genes could also augment resistance to As^V^ in other species or would, rather, require additional species-specific elements. To address this question, we expressed the *var* operon excluding the ArsR repressor in enteropathogens sensitive to As^V^ and studied their growth capacity in the presence of As^V^. Strikingly, induced coexpression of *varG-varH-arsJ* in several enteropathogens boosted their growth on As^V^ (Fig. 7B), demonstrating that the Var system is sufficient and compatible with the ArsCB system in providing resistance against As^V^ in other bacteria.

## DISCUSSION

Arsenic is ubiquitous in the environment due to natural geologic processes and environmental pollution (31, 32). Consequently, most living organisms, including bacteria, encode arsenic resistance proteins (23, 33–37). However, functional characterization of these systems and how they support bacterial fitness in environments with As^V^ is underappreciated. In this study, we describe that V. cholerae exhibits higher resistance to As^V^ than other enteric pathogens.

The strategy used by V. cholerae to resist As^V^ is different from that used by other gammaproteobacterial enteropathogens. While most of these species encode an ArsC arsenate reductase and an ArsB arsenite efflux pump, V. cholerae lacks the latter and has an inactive ArsC. Therefore, V. cholerae does not reduce As^V^ to expel it out of the cell. From an evolutionary perspective, this could be a vestige of a functional ArsCB system that, after losing the ArsB component, has inactivated ArsC to prevent the generation of highly toxic As^III^. It remains to be investigated whether V. cholerae ArsC has undergone functional diversification to fulfill another role in the cell, perhaps related to its neighboring hypothetical genes (*vc2164-vc2167*).

Our screening identified a four-gene operon (*var* operon) conferring resistance to V. cholerae that encoded the ArsR repressor, the As^V^ permease ArsJ, a putative GAPDH (VarG), and a phosphatase (VarH). Interestingly, while most ArsR derepress transcription by sensing As^III^ (38), the lack of As^III^ production in V. cholerae suggests that ArsR probably senses As^V^ in this bacterium. Although more research is required to determine ArsR responses to As^V^ and As^III^ in V. cholerae, comparative sequence analysis between V. cholerae ArsR and that of As^III^-producing bacteria have revealed variations in conserved residues, including the E→K and C→A substitutions in the As^III^ binding motif (38) (Fig. S7A).

10.1128/mbio.01654-22.7 FIG S7 The As^III^ binding site of ArsR is not conserved in V. cholerae, and *varH* is specific to the *Vibrionaceae* family. An ArsR amino acid sequence alignment from V. cholerae and representative enteropathogenic bacteria using Vector NTI software. ArsR cysteine-containing As^III^-binding motifs are depicted in the lower panel, with cysteine residues labeled in red. (B) Genomic organization comparison of the *var* operon in the *Vibrionaceae* family and representative bacteria done with Patric software (https://www.patricbrc.org/). The presence of *varH* is depicted in green in the dotted rectangle. Download FIG S7, TIF file, 1.9 MB. Copyright © 2022 Bueno et al. 2022 Bueno et al. https://creativecommons.org/licenses/by/4.0/ This content is distributed under the terms of the Creative Commons Attribution 4.0 International license.

Although GAPDH enzymes are mostly known for their role in glycolysis, they have been functionally linked to diverse processes such as autophagy, oxidative stress responses, apoptosis, posttranscriptional gene regulation, maintenance of DNA integrity, intracellular membrane trafficking, etc. (39–41). A previous study reported that the GAPDH homolog to V. cholerae’s VarG detoxified As^V^ by generating 1As3PG from G3P. However, such conclusions were drawn from *in vitro* experiments that used commercial glycolytic GAPDH from rabbits instead of the specific As^V^-inducible GAPDH and monitored As^V^ transport instead of direct 1As3PG detection (17). Our results, combining *in vitro* GAPDH activity assays and HRMS analyses from intracellular samples provided direct evidence that VarG is a GAPDH that preferentially uses As^V^ over P_i_ to synthesize 1As3PG. The role of VarG in V. cholerae’s As^V^ resistance adds to this growing list of GAPDH moonlighting functions.

Although the same study suggested that As^V^ is exported through ArsJ as 1As3PG (17), the more severe growth defect of V. cholerae’s *arsJ* compared to the *varG* mutant suggests that 1As3PG does not play a major role in the export of As^V^ in this bacterium. Accordingly, the *arsJ* mutant accumulates intracellular As^V^ ca. 6 times more than the *varG* mutant. Even if we cannot wholly discount the transport of 1As3PG through ArsJ (as this compound can nonetheless be produced in the absence of VarG, likely by the constitutive glycolytic Gap activity), the fact that we have not detected extracellular 1As3PG or increased 3PG in cultures supplemented with As^V^ suggests that ArsJ expels free As^V^.

During glycolysis, the GAPDH enzyme catalyzes the simultaneous phosphorylation and oxidation of G3P to form 1,3 bi-phosphoglycerate (1,3-BPG), which is further converted into 3PG by phosphoglycerate kinase coupled to the transfer of the high-energy phosphate to ADP, making ATP. We reasoned that while the metabolic complexing of As^V^ into 1As3PG might decrease its toxicity by reducing As^V^ interaction with V. cholerae’s proteome (Fig. 4D), the formation of 1As3PG could be a dead-end product that would affect a major energy-producing pathway. In line with this reasoning, our results showed that VarH presents low phosphatase activity compared to canonical phosphatases. As a low catalytic rate has been previously associated with enzymatic promiscuity (42), we hypothesized that the putative phosphatase VarH would function as an arsenatase that dissociates the 1As3PG complex to clear the glycolytic pathway and free As^V^ for its export through ArsJ. Indeed, inactivation of VarH results in increased intracellular levels of 1As3PG, which correlates with the accumulation of arsenate in the cell, thereby supporting the idea that ArsJ primarily exports free As^V^ in V. cholerae. Although synteny analyses suggest coevolution between ArsJ and VarG, the presence of VarH is rather exclusive to the *Vibrionaceae* (Fig. S7B). It remains to be investigated whether the amino acid differences in ArsJ between VarG^+^ and VarG^–^ species explain the choice of transporting free or complexed As^V^.

It has been previously reported that arsenic induces the production of ROS in the cell (43–45). Our results show that in the presence of As^V^, V. cholerae induces oxidative stress responses, i.e., elevated levels of the hydrogen peroxide-tolerance determinant PrxA (46) and multiple proteins involved in the synthesis of cysteine. As cysteine-containing proteins are targets of ROS (47), and cysteine is also the building block of glutathione, stimulating its synthesis might help to alleviate oxidative damage provoked by As^V^. Additionally, we found that As^V^ directly or indirectly (via ROS) alters the stability of proteins implicated in energy-generating pathways such as Tricarboxylic acid cycle (TCA), fermentation, and respiration. Indeed, As^V^ accumulation in the *var* mutant dissipated the cellular membrane potential (a proxy of the cell’s energy status). The correlation between As^V^ accumulation with ROS and membrane depolarization exhibited by the *var* mutants suggests that oxidative stress and energy depletion might account for their growth impairment in As^V^.

V. cholerae is a pathogenic bacterium endemic to Bangladesh and India, regions where the concentrations of As^V^ in water and soil are the highest worldwide, in some areas exceeding concentrations of 1 mM arsenic (48, 49). Hence, V. cholerae’s superior resistance to As^V^ could have evolved as an adaptive strategy to thrive in As^V^-rich environments while endowing the cholerae pathogen with a fitness advantage over free-living and host-associated neighboring bacteria. Interestingly, despite the phylogenetic distance, heterologous expression of the *var* operon enhanced As^V^ resistance in all the pathogens assayed, demonstrating that this system has a high degree of versatility to work in combination with other arsenic detoxification systems.

Collectively, this work proposes a novel mechanism for As^V^ detoxification in V. cholerae that is entirely independent of As^V^ reduction and As^III^ extrusion. Our results support a model in which the formation of a transient As^V^-containing glycolytic intermediate cushions the impact of free As^V^ in the cell. While a metabolic sink might temporarily aid cells in enduring As^V^ toxicity, this strategy may exert a high cost in the long term, as glycolysis is a major pathway in cell bioenergetics. We propose that “dearsenylation” of 1As3PG by VarH provides a double benefit by decongesting any jam that may occur in glycolysis and freeing As^V^ for extrusion to the extracellular medium by ArsJ.

The essentiality of the V. cholerae *var* operon to survive in environments with As^V^ (such as the host, where concentrations of 100 ppm As^V^ can be reached [50]) suggests that this machinery may be considered a novel class of bacterial target suitable for therapeutic intervention.

## MATERIALS AND METHODS

### Bacterial strains and growth conditions.

The strains used in this work are listed in Table S2. The V. cholerae strains used in this study are derivatives of the El Tor clinical isolate C6706. In addition to V. cholerae, this study also used the strains Escherichia coli K-12 MG1655 and the *arsC*::Transposon mutant strain from the Keio collection (51), enterohemorrhagic Escherichia coli EHEC O157:H7, Salmonella enterica serovar Typhimurium LT2, Citrobacter rodentium DBS100, Shigella flexneri M90T, and Yersinia pseudotuberculosis YPIII. Strains were grown aerobically at 37°C in 15-mL tubes containing 3 mL of complete LB medium (10 g tryptone, 10 g NaCl, and 5 g yeast extract/L). To grow Shigella flexneri, TSB medium was used (tryptone [pancreatic digest of casein], 17 g soytone [peptic digest of soybean], 3 g glucose, 2.5 g sodium chloride 5 g dipotassium phosphate 2.5). When used, Casamino Acids were supplemented at 1%. To study resistance to As^V^, 3 × 10^6^ overnight-grown cells were inoculated into 200 μL of LB medium with or without As^V^ at the indicated concentrations in 96-well plates, and optical density (OD) values were measured every 10 min using a BioTek Epoch 2 plate reader. Where appropriate, antibiotics were added to V. cholerae and E. coli cultures at the following concentrations: 200 (streptomycin, Sm), 100 (carbenicillin, Cb), 5 (chloramphenicol, Cm), and 50 μg/mL (kanamycin, Km).

10.1128/mbio.01654-22.9 TABLE S2 List of strains and plasmids used in this study. Download Table S2, DOCX file, 0.03 MB. Copyright © 2022 Bueno et al. 2022 Bueno et al. https://creativecommons.org/licenses/by/4.0/ This content is distributed under the terms of the Creative Commons Attribution 4.0 International license.

### Construction of plasmids to create V. cholerae mutants, overexpression constructs, and transcriptional fusions.

V. cholerae mutants were created by allelic exchange with the suicide plasmid pCVD442 (52). Upstream and downstream DNA fragments (ca. 1 kbp) flanking each coding region were PCR-amplified with the primers listed in Table S3. Upstream and downstream DNA fragments were spliced together by overlapping PCR. The resulting ca. 2-kb fragments were digested as indicated in Table S3 and cloned into pCVD442. For allelic exchange of V. cholerae *arsC* by E. coli *arsC*, a similar protocol was followed, but in this case the V. cholerae upstream and downstream DNA regions of V. cholerae *arsC* were spliced together with the E. coli *arsC* gene by overlapping PCR (see Tables S2 and S3). Constructs were transformed into E. coli DH5α λpir for amplification. They were confirmed by sequencing, transformed into the E. coli donor strain SM10 λpir, and conjugated for 6 h at 37°C with V. cholerae C6706 by mixing equal volumes (1 mL) of exponential-phase cultures and spot-plating. Single crossover V. cholerae organisms were selected on LB plates with Sm and Cb. Restreaked single colonies were then plated on salt-free LB agar containing 10% (wt/vol) sucrose and Sm. Colonies were streaked on carbenicillin plates to confirm loss of pCVD442 and were then checked by PCR for successful deletion mutants.

10.1128/mbio.01654-22.10 TABLE S3 List of primers used in this study. Download Table S3, DOCX file, 0.01 MB. Copyright © 2022 Bueno et al. 2022 Bueno et al. https://creativecommons.org/licenses/by/4.0/ This content is distributed under the terms of the Creative Commons Attribution 4.0 International license.

Overexpression of V. cholerae and E. coli genes was carried out by using the arabinose-inducible expression vectors pBAD33 and pBAD18,and the IPTG (isopropyl-β-d-thiogalactopyranoside)-inducible expression vector pHL100 (53) (see Table S2 for specific constructs used for each enteropathogen). To construct each overexpressing plasmid, gene open reading frames including ribosome binding sites were PCR amplified (see primers used in Table S3), double digested with the indicated restriction enzymes, and cloned into each plasmid. Constructs were transformed into the indicated strain by electroporation.

For construction of the transcriptional reporter fusions, gene promoter regions of target genes were amplified using the primers pairs indicated in Table S3. The PCR product was double digested with HindIII and EcoRI and then ligated into HindIII-EcoRI-digested pCB192N (54). Cloned plasmids were then transformed into V. cholerae by electroporation.

### Purification of recombinant VarG, VC2000, VC1041, and VarH.

V. cholerae VarG, VC1041, and VarH proteins were overexpressed using the E. coli strain BL21 as 6× His-tagged enzymes from a pET28b vector. Overnight cultures were diluted into 250 mL LB broth and grown until an OD at 600 nm (OD_600_) of 0.5. Flasks were supplemented with 1 mM IPTG and shaken at 37°C for 2 h. Cells were pelleted and resuspended in equilibration buffer (50 mM Tris, pH 7.5, 50 mM NaCl, with protease inhibitor cocktail [Roche]) and disrupted by passaging once through a French press. Lysates were then centrifuged for 1 h (25,000 rpm, Beckman Coulter Avanti J26-XP centrifuge, JL-25.50 rotor) at 4°C. Nickel-NTA resin (0.5 mL resuspended in equilibration buffer) was then added to the supernatant, followed by incubation at 4°C in a rotating wheel. The lysate was separated from the resin by centrifugation for 1 min at 3,220 × *g*, and the resin was washed (5 × 10 mL) with washing buffer (equilibration buffer adjusted to 1 M NaCl) and eluted with 2 mL of washing buffer containing 500 mM imidazole. Fractions were subjected to SDS-PAGE and Coomassie brilliant blue staining for purity assessment. Protein concentration was quantified with a Bradford assay.

### Proteomic and thermal proteome profiling (TPP).

We performed TPP to determine protein abundance and thermal stability changes in WT and *var* mutant strains upon exposure to As^V^ similarly to what was previously described (30, 55). Briefly, WT and mutant cells grown to an OD_578_ of 0.5 were incubated with 1 mM arsenate (or a similar volume of water) for 10 min. Cells were then washed with 10 mL phosphate-buffered saline (PBS) (for cells treated with arsenate, 1 mM arsenate was included in the wash step) and aliquoted to a PCR plate. Each aliquot was then exposed to a different temperature in the range of 37 to 66.3°C for 3 min. Following a 3-min incubation at room temperature, cells were lysed with lysis buffer (final concentration, 50 μg mL^−1^ lysozyme, 0.8% NP-40, 1× protease inhibitor [Roche], 250 U mL^−1^ benzonase, and 1 mM MgCl_2_ in PBS) for 20 min, followed by three freeze-thaw cycles. Lysates were then prepared for analysis by mass spectrometry by digesting proteins using a modified solid-phase-enhanced sample-preparation (SP3) protocol (56), labeling peptides with TMTpro (Thermo Fisher Scientific) and pooling samples from the same temperature together. These samples were then fractionated to six fractions with high-pH fractionation and injected on an Orbitrap Q-Exactive Plus instrument (Thermo Fisher Scientific) coupled to liquid chromatography (details on the run conditions and instrument parameters as described in Mateus et al. (30, 55)).

Mass spectrometry raw data were searched against the V. cholerae FASTA file (UP000000584, downloaded from UniProt) using the Mascot 2.4 (Matrix Science) search engine and isobarquant (57). Protein abundance and thermal stability changes were determined using Limma (58) using the same algorithm as in Mateus et al. (55).

### Phosphorylation state of VarG.

To identify the VC1069 GAPDH phosphorylation state, V. cholerae wild-type and Δ*varH* strains carrying the overexpression vector pHL100 containing the *varG*-HIS tagged gene clone were grown in the presence of As^V^ and with IPTG. Cells were pelleted, disrupted, and loaded and run in SDS-PAGE gel. VarG-HIS bands were excised and diluted with NuPAGE LDS sample buffer (Invitrogen, Carlsbad, CA) mixed with 10 mM dithiothreitol (DTT) and incubated at 56°C for 20 min, followed by addition of iodoacetamide (IAA) to a final concentration of 20 mM. The sample was loaded on 4 to 12% NuPAGE (Invitrogen). The electrophoresis was run in MOPS (morpholinepropanesulfonic acid) buffer at 180 V for 1 h. The gel was stained with Coomassie brilliant blue. Bands of interest were cut from the SDS-PAGE. The bands were digested with trypsin, followed by identification using liquid chromatography-tandem mass spectrometry (LC-MS/MS). MS/MS spectra were searched with Proteome Discoverer 2.3 (Thermo Fisher Scientific) against the two sequences obtained for phosphate groups (UniProtKB). The precursor tolerance and fragment tolerance were set to 10 ppm and 0.05 Da, respectively. Trypsin was selected as the enzyme, and methionine oxidation, phosphorylation of serine, tyrosine, and tryptophan, and deamidation of asparagine and glutamine were treated as dynamic modifications, and carbamidomethylation of cysteine was treated as a fixed modification.

### Transposon-insertion sequencing (TIS) analysis.

TIS was carried out essentially as previously described (59) but using selection plates containing LB medium with As^V^ 1 mM. In brief, ~600,000 transposon mutants were generated by conjugation of V. cholerae C6706 with SM10λ*pir* E. coli carrying the Himar1 suicide transposon vector pSC189 (60). They were collected, and their genomic DNA was pooled and analyzed on a MiSeq benchtop sequencer (Illumina, San Diego, CA). Insertion sites (which included 35% of TA sites) were identified as previously described (59), and significance was determined using Con-ARTIST simulation-based normalization as described (61). Results were visualized using Artemis (62).

### As^V^ resistance screen of arrayed transposon mutant library.

A nonredundant transposon insertion library from the V. cholerae strain C6706 (63) consisting of 3,156 insertion mutants was used to determine genetic determinants providing resistance to As^V^ in V. cholerae. V. cholerae WT and the library insertion mutants were first grown aerobically in 200 μL of LB in 96-well plates at 37°C for 24 h. These cultures were then used as inoculum in 96-well plates containing 200 μL complete LB medium, in the presence of 1 mM As^V^. The 96-well plates were incubated under agitation at 37°C for 24 h, and then resistance to As^V^ was evaluated by measuring the optical density (OD_600_) using a BioTek Eon plate reader.

### Protein homology studies of As^V^ resistance determinants in enteric pathogens.

DNA sequences of the V. cholerae *arsR*, *varG*, *varH*, and *arsJ var* genes and complete genomes sequences of the enteric pathogens Salmonella Typhimurium (BioProject no. PRJNA241), Citrobacter rodentium (PRJNA261533), Shigella flexneri (PRJNA553848), EHEC (PRJNA218110), and Yersinia pseudotuberculosis (PRJEB6403) were downloaded from the NCBI database. Complete genomes of the pathogens were translated using Prodigal ver. 2.6.2, and homologous sequences to the V. cholerae Var proteins were queried against the enteric pathogens by BLASTP ver. 2.9.0. Sequence homology data were represented in a heatmap diagram with Spearman correlation done with R ver. 3.6.1.

### β-Galactosidase activity determination.

β-Galactosidase activity was measured through *o*-nitrophenyl-β-d-galactopyranoside (ONPG) cleavage by the product of the *lacZ* reporter gene, and specific activity was calculated in Miller units (64). In brief, cultures of V. cholerae carrying the pCB192N plasmid harboring promoter gene insertions (see Table S2) were grown at 37°C overnight in 15-mL Falcon tubes containing 3 mL of LB complete medium. Overnight cultures were diluted 1:200 and supplemented with 1 mM As^V^ and incubated at 37°C until they reached mid-log phase (OD_600_, 0.4 to 0.7). Aliquots (100- μL) of three different subcultures were collected, and cells were permeabilized and assayed in triplicate for each strain as previously described (64).

### Determination of intracellular and extracellular As^V^ and As^III^ in V. cholerae.

To determinate concentrations of As^V^ and As^III^ in supernatants of V. cholerae and E. coli, bacterial cells were grown in the presence of As^V^ for 5 h and then pelleted. Supernatants of each strain were identified to the species level by high-performance liquid chromatography (HPCL) (Agilent Infinity II 1290) using a C_18_ column reverse-phase method (130 Å, 1.7 μm, 2.1 mm by 150 mm; Waters, USA). The elution conditions used were flow rate, 1 mL min^−1^; temperature, 25°C; isocratic elution in 5 mM tetrabutylammonium hydroxide, 5% methanol (vol/vol) and 3 mM malonic acid. Identification and quantification of As^V^ and As^III^ were performed by inductively coupled plasma mass spectroscopy (ICP-MS) (Agilent 8900 ICP-MS Triple Quad) by comparison to standards of known concentration and peak integration.

To determine intracellular concentrations of As^V^ and As^III^ in V. cholerae, the pelleted cells were washed in complete LB medium to remove traces of arsenic and then resuspended in 500 μL of H_2_O. Glass beads were adding to the resuspension, and cells were lysed by bead-beating. The resulting extract was centrifuged, and As^V^ and As^III^ from those supernatants were analyzed by ICP-MS as previously described. Samples were analyzed in triplicate.

### LC-MS/MS analysis using the QTRAP 6500+.

To measure 3-phosphoglycerate (3PG), V. cholerae cells grown in the presence of 5 mM As^V^ were pelleted and used for LC-MS/MS analysis. Bacterial pellets were resuspended in 1 mL ice-cold 60% ethanol containing 2 μM ribitol as the internal control. The cells were mechanically disrupted using a bead homogenizer set to oscillate for 3 cycles of 30 s each at 6,800 rpm with a 10-s pause between each cycle. Cell debris was separated at 13,000 rpm. An aliquot (200 μL) of the supernatant containing intracellular metabolites was vacuum dried and resuspended in an equal volume of 7.5 mM ammonium bicarbonate solution before LC-MS/MS analysis.

A triple-quadrupole-ion trap hybrid mass spectrometer, QTRAP 6500+ instrument (SCIEX, USA) connected with a Waters ultraperformance liquid chromatography (UPLC) I-class system was used for metabolite analysis. The chromatographic separation was performed on an XSELECT HSS XP column (150 mm by 2.1 mm inside diameter [i.d.]; 2.5 μm particle size; Waters, USA) using a binary solvent system at a flow rate of 0.1 mL/min. Mobile phase A was composed of 7.5 mM ammonium bicarbonate in LC-MS-grade water, and mobile phase B was 100% LC-MS grade methanol. The column was maintained at 40°C, and the autosampler temperature was set to 7°C. The A/B solvent ratio was maintained at 100/0 for 2 min, followed by a gradual increase of solvent B to 95% for 2 min. The solvent B was maintained at 95% over the next 5 min. The gradient was reduced to 100% solvent A within 0.5 min, and the column was equilibrated for 5.5 min before the next run. The needle was washed with 1.2 mL of a strong wash solution containing 100% LC-MS-grade acetonitrile followed by 1 mL of a weak wash solution composed of 10% aqueous methanol before each injection. The injection volume was 5 μL. The QTRAP 6500+ (SCIEX) was operated in negative as well as positive ion mode for targeted quantitation in multiple reaction monitoring (MRM). MRM parameters for each analyte are listed in Table 1. The electrospray ionization (ESI) parameters used are as follows: electrospray ion voltage of −4,500 V in negative and 5,500 V in positive mode, source temperature of 400°C, curtain gas of 35, and gas 1 and 2 of 40 lb/in^2^ each. The compound-specific parameters, such as declustering potential (DP) and collision energy (CE), were optimized for each compound using manual tuning. These values are listed in Table 1.

**TABLE 1:** MRM transitions, declustering potentials, and collision energies of metabolites

Analyte	Q1 (m/z)	Q3 (m/z)	DP (V)	CE (V)
3-Phospho glycerate	187.2	98.9	+60	19.2
Ribitol	150.8	88.9	−60	−14.2

### Detection of 1As3PG.

1As3PG was identified using a Thermo Orbitrap Exploris 480 high-resolution mass spectrometer. The chromatographic method used for this analysis was the same as that described for the LC-MS/MS performed using the QTRAP6500+. The MS data were acquired in both positive and negative polarity within mass range of 140 to 400 Da. The precursor ion resolution was maintained at 240,000, whereas the product ion resolution was maintained at 120,000. Deprotonated [M-H]^–^ ion of 1As3PG was detected at *m/z* 304.8696 Da compared to the actual mass of 304.8674 Da; thus, Δm = 7.149 ppm. The fragmentation pattern of 1As3PG could not be further resolved under these conditions.

### Protein structural and topological analysis.

Protein alignments were performed by using Vector NTI. Prediction of the VarH secondary structure was performed by using Phyre2 (http://www.sbg.bio.ic.ac.uk/phyre2/html/page.cgi?id=index). Structural prediction of the VarG and VarH proteins was performed with AlphaFold2 on the CoLabFold publicly accessible interface (65) (https://colab.research.google.com/github/sokrypton/ColabFold/blob/main/AlphaFold2.ipynb). Sequences were modeled as monomers using mmseqs2 for multiple sequence alignment. Studies on protein topological similarity were carried out by using TM-score (https://zhanggroup.org/TM-score/).

### Competition assays.

Aerobic overnight cultures of V. cholerae C6706 wild-type *lacZ*^–^ and enterohemorrhagic Escherichia coli EHEC O157:H7 *lacZ^+^*, Salmonella enterica serovar Typhimurium *LT2 lacZ^+^*, Citrobacter rodentium DBS100 *lacZ^+^*, Shigella flexneri M90T *lacZ^+^*, and Yersinia pseudotuberculosis YPIII *lacZ^+^* were collected, and 1 × 10^7^ cells were coinoculated at a 1:1 ratio in 15-mL tubes containing 3 mL of complete LB medium without or with 10 mM As^V^. After 8 h of incubation at 37°C, an aliquot was diluted and plated on LB agar plates containing X-Gal (5-bromo-4-chloro-3-indolyl-β-d-galactopyranoside) to enumerate wild-type and competing strains. Competitive indices (CIs) were determined by dividing the ratio mutant or non-WT strain to C6706 wild-type colonies by the ratio in the inoculum. To calculate CIs among V. cholerae strain C6706 against a different V. cholerae strain against enteric pathogens (Fig. 1B), CIs obtained after the As^V^ challenge were normalized to the respective CIs obtained after competition in the absence of As^V^.

### *In vitro* GAPDH and phosphatase enzymatic assays.

The GAPDH activity assay was performed in accordance with the manufacturer’s instructions (MAK27; Sigma-Aldrich) but using P_i_ or As^V^ as the GAPDH substrate; 10 μg of purified V. cholerae VarG and VC2000 enzymes were used in the assay. This assay quantifies GAPDH activity by measuring enzymes’ capacity to reduce NAD^+^ to NADH during the conversion of glyceraldehyde 3-phosphate to 1,3-biphosphoglycerate. The NADH produced in this reaction results in a colorimetric (OD_450_) product proportional to the GAPDH activity of the enzyme. GAPDH activity is reported as nmol/min/mL = milliunit/mL. One unit of GAPDH is the amount of enzyme that will generate 1.0 mmol of NADH per minute at pH 7.2 at 37°C.

Phosphatase activity was quantified using Cayman’s phosphatase colorimetric assay kit and 5 μg of purified V. cholerae VarH, C113G R119G VarH, VC1041 (positive control), and BsrV (negative control) enzymes. The assay uses *p*-nitrophenylphosphate (pNPP) as the substrate for phosphatase enzymes. Phosphatase enzymes dephosphorylate pNPP, which deprotonates its phenolic OH-group by increasing the pH of the reaction; deprotonated pNP yields an intense yellow color detected at OD 405 nm. Enzymatic activities were calculated as μmol of phosphate released per min/mL.

### Flow cytometry analysis.

V. cholerae WT and the indicated *var* mutant strains were grown for 5 h in the presence of 1 mM As^V^, and then aliquots (1 mL) from each culture were pelleted, washed once, and resuspended in PBS (100 μL) to measure membrane potential and ROS.

To measure membrane potential in V. cholerae cells, we used the BacLight bacterial membrane potential kit (Invitrogen). V. cholerae samples resuspended in 100 μL PBS were incubated for 15 min at 20°C in the dark with the dye 30 μM 3,3′-diethyloxacarbocyanine iodide (DiOC_2_). Next, cells were washed twice with PBS and resuspended in 2 mL of PBS, and then the fluorescence emitted from treated cells was measured with a Bio-Rad S3e cell sorter at an excitation wavelength of 488 nm and an emission wavelength of 525/30 nm (for green) or 655 nm (for red).

ROS measurements were performed using the CellROX green reagent for oxidative stress detection (Thermo Fisher). Washed V. cholerae cells were supplemented with CellROX reagent at a final concentration of 5 μM and were incubated for 30 min at 37°C. Next, cells were washed twice with PBS and resuspended in 2 mL of PBS, and then the fluorescence emitted was measured with a Bio-Rad S3e cell sorter at an excitation wavelength of 488 nm and an emission wavelength of 525 nm.

### Data availability.

The TIS raw data can be found at https://data.mendeley.com/datasets/kj9xxffpc7/1. A table showing underrepresented genes found by TIS can be found at https://data.mendeley.com/datasets/c64h244vs8/2. Proteomic and TPP raw data can be found at http://www.ebi.ac.uk/pride/archive/projects/PXD035237 and ftp://ftp.pride.ebi.ac.uk/pride/data/archive/2022/07/PXD035237. A table showing TPP targets can be found at https://data.mendeley.com/datasets/rhp3fs33yy/2.

### Statistics and reproducibility.

Statistical significance was assessed with Student’s *t* test where indicated. A *P* value of less than 0.05 was considered statistically significant. Assays were performed with three biological replicates unless otherwise indicated.
