# Supplementary material for: Transient Glycolytic Complexation of Arsenate Enhances Resistance in the Enteropathogen Vibrio cholerae
Source: mBio. 2022 Sep 14;13(5):e01654-22. doi: 10.1128/mbio.01654-22 (PMC9601151; doi:10.1128/mbio.01654-22)
Supplement: TABLE S1 [file mbio.01654-22-s0008.docx]

**Table S1.** **Complete list of genes identified under-represented by TIS in the presence of 1 mM As^V^ with thresholds of fold change <0.1 and an inverse p-value >75**

| **locus** |  | **ratio LB (+As^V^/-As^V^)** | **inv p-val >100 (p<0.01)** |
| --- | --- | --- | --- |
| *vc1033* | zinc/cadmium/mercury/lead-transporting ATPase | 0,008305558 | 6,25696E+11 |
| *vc0468* | glutathione synthetase | 0,01173324 | 20755,11276 |
| *vc1716* | condesin subunit F | 0,013628702 | 16719,39865 |
| *vc2266* | thiamine monophosphate kinase | 0,013913404 | 37207,40702 |
| *vc1682* | peptide ABC transporter permease | 0,014064954 | 82300,28842 |
| *vc2465* | periplasmic negative regulator of sigmaE | 0,014171923 | 45019,43809 |
| *vc1715* | condesin subunit E | 0,01484158 | 691,8777885 |
| *vc1069* | glyceraldehyde-3-phosphate dehydrogenase | 0,016154243 | 11388096,21 |
| *vc1421* | hypothetical protein | 0,01835443 | 15384,04244 |
| *vc1837* | TolA protein | 0,022150823 | 2243,731015 |
| *vc1839* | TolQ protein | 0,022400304 | 644,0558693 |
| *IG_VC1449* |  | 0,022909354 | 149,8907288 |
| *vc1836* | translocation protein TolB | 0,022989166 | 9362,187734 |
| *vc0581* | lipoprotein | 0,023076239 | 31393578,54 |
| *vc0941* | serine hydroxymethyltransferase | 0,026475713 | 2122,108598 |
| *vc2655* | lysyl-tRNA synthetase | 0,026737288 | 461,2780263 |
| *vc1619* | hypothetical protein | 0,029459002 | 456,4091098 |
| *vc2661* | hypothetical protein | 0,030461713 | 129,7314404 |
| *vc0958* | apolipoprotein N-acyltransferase | 0,032399718 | 16146995,06 |
| *vc1730* | DNA topoisomerase I | 0,040842768 | 111,4375197 |
| *vc1714* | cell division protein MukB | 0,041129576 | 123149966,2 |
| *vc1071* | transporter | 0,041617105 | 2069678,123 |
| *vc0212* | lipid A biosynthesis (KDO)2-(lauroyl)-lipid IVA acyltransferase | 0,042209136 | 26385,62212 |
| *vc1449* | hypothetical protein | 0,049628334 | 117,8151474 |
| *vc0745* | inositol monophosphatase | 0,05025158 | 109,0097914 |
| *vc0225* | lipopolysaccharide biosynthesis protein | 0,050739032 | 2836,907317 |
| *vc0547* | aspartate kinase | 0,052946814 | 58123838,93 |
| *vc1070* | phosphatase | 0,053713413 | 693,095589 |
| *vc0633* | outer membrane protein OmpU | 0,059148501 | 3003542,783 |
| *vc2414* | pyruvate dehydrogenase subunit E1 | 0,062526783 | 8292292175 |
| *vc1683* | peptide ABC transporter ATP-binding protein | 0,064171919 | 359,8109618 |
| *vc0911* | trehalose-6-phosphate hydrolase | 0,068093172 | 334868,4631 |
| *vc2040* | hypothetical protein | 0,068539958 | 126,9688536 |
| *vc2295* | Na(+)-translocating NADH-quinone reductase subunit A | 0,074385638 | 3311124,135 |
| *vc2635* | penicillin-binding protein 1A | 0,074889792 | 1121279,769 |
| *vc1887* | hypothetical protein | 0,079807502 | 959,1616461 |
| *vc1491* | dihydroorotate dehydrogenase 2 | 0,080045128 | 1476,948406 |
| *vc2293* | Na(+)-translocating NADH-quinone reductase subunit C | 0,088732015 | 3512,09842 |
| *vc1358* | hypothetical protein | 0,094189582 | 115,7288862 |
| *vc1730* | DNA topoisomerase I | 0,040842768 | 111,4375197 |
| *vc0745* | inositol monophosphatase | 0,05025158 | 109,0097914 |
| *vc0848* | SsrA-binding protein | 0,068003435 | 96,10522624 |
| *vc0576* | stringent starvation protein A | 0,03158335 | 78,96453327 |
| *vc2625* | ribulose-phosphate 3-epimerase | 0,057927689 | 78,37968913 |
