# Supplementary material for: Transient Glycolytic Complexation of Arsenate Enhances Resistance in the Enteropathogen Vibrio cholerae
Source: mBio. 2022 Sep 14;13(5):e01654-22. doi: 10.1128/mbio.01654-22 (PMC9601151; doi:10.1128/mbio.01654-22)
Supplement: TABLE S2 [file mbio.01654-22-s0009.docx]

**Table S2. List of strains and plasmids used in this study.**

|  | **Relevant genotype or description** | **References or source** |
| --- | --- | --- |
| ***Escherichia coli*** | | |
| DH5α | Cloning strain | Cava Lab stock |
| DH5α *λpir* | Cloning strain, pir R6K | Cava Lab stock |
| SM10 *λ*pir | *thi thr leu tonA lacY supE* *recA*::RP-4-2-Tc::Mu(lpir) *pro* *endA hsdA hsdR supF* | (1) |
| K-12 MG1655 | F- lambda- *ilvG*- *rfb*-50 *rph*-1 | Cava Lab stock |
| *arsC*::Tn | *E. coli* Keio mutant *arsC*, Km^r^ | (2) |
| *arsC* Tn::-*pBAD33Vc.arsC* | *E. coli* Keio *arsC*::Tn overexpressing *V. cholerae arsC gene*, Km^r^ Cm^r^ | This study |
| *arsC* Tn::-*pBAD33Ec.arsC* | *E. coli* Keio *arsC*::Tn overexpressing *E.coli arsC gene*, Km^r^ Cm^r^ | This study |
| EHEC O157:H7 | Wild-type EHEC *serotype* O104:H4 | Matthew K.Waldor Lab stock |
| ***Salmonella enterica*** |  |  |
| LT2 | Wild-type isolate of *S. enterica* subsp. enterica serovar Typhimurium | ATCC 14028 |
| LT2  pBAD33*varG-arsJ* | Wild-type isolate of *S. enterica* subsp. enterica serovar Typhimurium carrying the *V. cholerae* genes *varG-varH-arsJ* cloned into the overexpression vector pBAD33. | This study |
| ***Citrobacter rodentium*** |  |  |
| DBS100 | Wild-type | Matthew K.Waldor Lab stock |
| DBS100  pHL100*varG-arsJ* | Wild-type isolate of *C. rodentium* DBS100 carrying the *V. cholerae* operon *varG-varH-arsJ* cloned into the overexpression vector pHL100. | This study |
| ***Shigella flexneri*** |  |  |
| M90T | Wild-type | (3) |
| M90T  pBAD18*varG-arsJ* | Wild-type isolate of *S. flexneri* M90T carrying the *V. cholerae* operon *varG-varH-arsJ* cloned into the overexpression vector pBAD18. | This study |
| ***Yersinia pseudotuberculosis*** |  |  |
| YPIII | Wild-type | Caliper Life Sciences, Inc. |
| YPIII *pBAD33varG-arsJ* | Wild-type *Yersinia pseudotuberculosis* YPIII carrying the *V. cholerae* operon *varG-varH-arsJ* cloned into the overexpression vector pBAD33. | This study |
| ***Vibrio cholerae*** | | |
| C6706 | Wild-type C6706 lacZ^-^, Sm^r^ | Cava Lab stock |
| C6706 | Wild-type C6706 lacZ^+^, Sm^r^ | Cava Lab stock |
| ∆*arsR* | *V. cholerae* C6706 ∆*arsR,* Sm^r^ | This study |
| ∆*varG* | *V. cholerae* C6706 ∆*varG,* Sm^r^ | This study |
| ∆*varH* | *V. cholerae* C6706 ∆*varH,* Sm^r^ | This study |
| ∆*arsJ* | *V. cholerae* C6706 ∆*arsJ,* Sm^r^ | This study |
| ∆*varG*∆*varH* | *V. cholerae* C6706 ∆*varG*∆*varH,* Sm^r^ | This study |
| ∆*varG*∆*varH*∆*arsJ* | *V. cholerae* C6706 ∆*varG*∆*varH*∆*arsJ,* Sm^r^ | This study |
| ∆*varH*∆*arsJ* | *V. cholerae* C6706 ∆*varH*∆*arsJ,* Sm^r^ | This study |
| ∆*arsC* | *V. cholerae* C6706 ∆*arsC,* Sm^r^ | This study |
| *arsC^vc^/arsC^ec^* | *V. cholerae* C6706 where the *arsC* gene has been replaced by the *arsC E. coli.* | This study |
| *arsC^vc^/arsC^ec^ + pH100arsB^ec^* | *V. cholerae* C6706 *Vc arsC^vc^/arsC^ec^* strain where the *arsB* gene from *E. coli* is overexpressed in trans from the pHL100 vector, Km^r^ | This study |
| pHL100*varG-*HIS | *V. cholerae* C6706 carrying the *varG* gene tagged with a His tag in the C- end and cloned into the overexpression vector pHL100, Km^r^ | This study |
| ∆*vc1070*pHL100*varG-*HIS | *V. cholerae* C6706 *∆varH* mutant strain carrying the *varG* gene tagged with a histidine tag in the C- end and cloned into the overexpression vector pHL100, Km^r^ | This study |
| pCB192Np*arsR* | *V. cholerae* C6706 carrying the pCB192N plasmid containing the *arsR* gene promoter fused to the *lacZ* expression reporter gene, Cb^r^ | This study |
| pCB192Np*varG* | *V. cholerae* C6706 carrying the pCB192N plasmid containing the *varG* gene promoter fused to the *lacZ* expression reporter gene, Cb^r^ | This study |
| pCB192Np*varH* | *V. cholerae* C6706 carrying the pCB192N plasmid containing the *varH* gene promoter fused to the *lacZ* expression reporter gene, Cb^r^ | This study |
| pCB192Np*arsJ* | *V. cholerae* C6706 carrying the pCB192N plasmid containing the *arsJ* gene promoter fused to the *lacZ* expression reporter gene, Cb^r^ | This study |
| **Plasmids** | | |
| pCVD442 | Suicide vector containing the counter-selectable marker sacB (Ap^R^) | (4) |
| pCVD442Δ*arsR* | pCVD442 carrying upstream and downstream regions of *arsR* | This study |
| pCVD442Δ*varG* | pCVD442 carrying upstream and downstream regions of *varG* | This study |
| pCVD442Δ*varH* | pCVD442 carrying upstream and downstream regions of *varH* | This study |
| pCVD442Δ*arsJ* | pCVD442 carrying upstream and downstream regions of *arsJ* | This study |
| pCVD442Δ*vc2164* | pCVD442 carrying upstream and downstream regions of *vc2164* | This study |
| pCVD442Δ*arsC* | pCVD442 carrying upstream and downstream regions of *arsC* | This study |
| pCVD442Δ*arsC^vc^- arsC^ec^* | pCVD442 carrying the *arsC* gene from *E. coli* with the 5´ and 3´end termini linked to the upstream and downstream regions of the *arsC* gene, respectively, from *V. cholerae* | This study |
| pCVD442*arsJ0C113G-R119G* | pCVD442 carrying the *varH* gene where nucleotides coding to Cys113 and Arg119 have been replaced by nucleotides coding by Gly | This study |
| pSC189 | Sm10λPIR pSC189, Km^r^ | (5) |
| pBAD33 | Replicating plasmid used for expressing genes under control of the arabinose-inducible P*ara* promoter; Cm^R^ | (6) |
| pBAD18 | Replicating plasmid used for expressing genes under control of the arabinose-inducible P*ara* promoter; Km^r^ | (6) |
| pHL100 | Replicating plasmid used for expressing genes under control of the IPTG-inducible P*lac* promoter; Km^R^ | (7) |
| pBAD33*varGvarHarsJ* | pBAD33 containing the *varG*, *varH* and *arsJ* genes. | This study |
| pBAD18 *varGvarHarsJ* | pBAD18 containing the *varG*, *varH* and *arsJ* genes. | This study |
| pH100 *varGvarHarsJ* | pHL100 containing the *varG*, *varH* and *arsJ* genes. | This study |
| pH100*varG-*HIS | pHL100 containing the *varG* gene carrying a His-tag at its C- end. | This study |
| pCB192N | β-galactosidase promoter-probe vector; Ap^R^ | (7) |
| pCB192Np*arsR* | pCB192N containing the *arsR* promoter gene | This study |
| pCB192Np*varG* | pCB192N containing the *varG* promoter gene | This study |
| pCB192Np*varH* | pCB192N containing the *varH* promoter gene | This study |
| pCB192Np*arsJ* | pCB192N containing the *arsJ* promoter gene | This study |
| Pet28b | Replicating plasmid for protein overexpression | Novagen |
| Pet28b*varG* | Overexpression plasmid pET28b containing the *varG* gene | This study |
| Pet28b*varH* | Overexpression plasmid pET28b containing the *varH* gene | This study |
| Pet28b*Gap* | Overexpression plasmid pET28b containing the *Gap* gene | This study |
| Pet28b*varHC113G-R119G* | Overexpression plasmid pET28b containing the *varH* gene where the Cys113 and Arg119 have been replaced by Gly. | This study |

**References**

1. Miller VL, Mekalanos JJ. 1988. A novel suicide vector and its use in construction of insertion mutations: Osmoregulation of outer membrane proteins and virulence determinants in Vibrio cholerae requires toxR. J Bacteriol 170:2575–2583.

2. Baba T, Ara T, Hasegawa M, Takai Y, Okumura Y, Baba M, Datsenko KA, Tomita M, Wanner BL, Mori H. 2006. Construction of Escherichia coli K-12 in-frame, single-gene knockout mutants: The Keio collection. Mol Syst Biol 2:1–11.

3. Sansonetti PJ, Kopecko DJ, Formal SB. 1982. Involvement of a Plasmid in the Invasive Ability of Shigella flexneri 35:852–860.

4. Donnenberg MS, Kaper JB. 1991. Construction of an eae deletion mutant of enteropathogenic Escherichia coli by using a positive-selection suicide vector. Infect Immun 59:4310–7.

5. Chiang SL, Rubin EJ. 2002. Construction of a mariner-based transposon for epitope-tagging and genomic targeting. Gene 296:179–185.

6. Guzman L, Belin D, Carson MJ. 1995. Tight Regulation, Modulation, and High-Level Expression by Vectors Containing the Arabinose PBAD Promoter. J Bacteriol 177:4121–4130.

7. Kimsey HH, Waldor MK. 2009. Vibrio cholerae LexA coordinates CTX prophage gene expression. J Bacteriol 191:6788–6795.
