# Supplementary material for: Transient Glycolytic Complexation of Arsenate Enhances Resistance in the Enteropathogen Vibrio cholerae
Source: mBio. 2022 Sep 14;13(5):e01654-22. doi: 10.1128/mbio.01654-22 (PMC9601151; doi:10.1128/mbio.01654-22)
Supplement: TABLE S3 [file mbio.01654-22-s0010.docx]

**Table S3. List of primers used in this study.**

| **Primer name** | **DNA sequence (5´ to 3´)** | **Additional sequences added*** |
| --- | --- | --- |
| ∆*arsR* | CTGACTCTAGATTGGTATTGCTAAGAATCGTG | XbaI cut site |
|  | TTTTTTTGCGGCCGCTTTTTTAATCAAACTCCAATATATATG | Linker for OP |
|  | AAAAAAGCGGCCGCAAAAAAAAGAACCAAGTTGAGTTATCCC | Linker for OP |
|  | CTGACTCTAGAGATGAGGCTCATACCGCAAGC | XbaI cut site |
| ∆*varG* | CTGACTCTAGATTGGTATTGCTAAGAATCGTG | XbaI cut site |
|  | TTTTTTTGCGGCCGCTTTTTTACGCCCAATACGACCAAATCC | Linker for OP |
|  | AAAAAAGCGGCCGCAAAAAAAGGTTACGCCACGCGCACCGCG | Linker for OP |
|  | CTGACTCTAGAACCTTTTAAGGCATTCTTTGA | XbaI cut site |
| ∆*varH* | CTGACTCTAGAGTGATTGAGGCTACGGGTAAG | XbaI cut site |
|  | TTTTTTTGCGGCCGCTTTTTTTTTTTACTACTCCTTATGCCAAG | Linker for OP |
|  | AAAAAAGCGGCCGCAAAAAAACCCGATCAAGGGCCGCATAAG | Linker for OP |
|  | CTGACTCTAGACGCCCACAGCACTGTGATCCC | XbaI cut site |
| ∆*arsJ* | CTGACTCTAGAACTGGGTATCGCACAAGCCTC | XbaI cut site |
|  | TTTTTTTGCGGCCGCTTTTTTCACGAGCATGTACTGACGAAC | Linker for OP |
|  | AAAAAAGCGGCCGCAAAAAAACTGATCTCATTTTGGCTGCCG | Linker for OP |
|  | CTGACTCTAGACTTCACCATCGGTTTCCAGAG | XbaI cut site |
| ∆*arsC* | CTGACTCTAGATACCAGAAGACCGGATTACCG | XbaI cut site |
|  | TTTTTTTGCGGCCGCTTTTTTTGACATAACTTCTCCTGTATC | Linker for OP |
|  | AAAAAAGCGGCCGCAAAAAAAATTCTATGATTTCGATAGTGG | Linker for OP |
|  | CTGACTCTAGAGATGAGGCTCATACCGCAAGC | XbaI cut site |
| *arsC^vc^/arsC^ec^* | GCTAGGTCGACGATTAACTACACGCGTAGCAATG | SalI cut site |
|  | GATAAATGGTAATGTTGCTCATAACTTCTCCTGTATCAGATTTA | OP primer 1 |
|  | TAAATCTGATACAGGAGAAGTTATGAGCAACATTACCATTTATC | OP primer 2 |
|  | TAATAGAGCACCACTATCGAAATTATTTCAGGCGCTTACCCGC | OP primer 3 |
|  | GCGGGTAAGCGCCTGAAATAATTTCGATAGTGGTGCTCTATTA | OP primer 4 |
|  | GCTAGTCTAGAAGCTTTAAGCCTAACTCCTTAC | XbaI cut site |
| Pet28b *varHC113G-R119G-* | CGCCATATGACGCATCCAACTTGGGAATTAC | NdeI cut site |
|  | GTAAGCCAGTTCCACCCGAACCTCCCATTCCATGCAAAGCGAC | OP primers with C113 and R119 replaced by Gly |
|  | GTCGCTTTGCATGGAATGGGAGGTTCGGGTGGAACTGGCTTAC |  |
|  | TCGGAATTCGGTTTGACGATTTGATGCACG | EcoRI cut site |
| pBAD33*arsC^vc^* | GCTAGTCTAGAAGTTAATGGTGCAACGCGAGCGC | XbaI cut site |
|  | GCTAGAAGCTTTCAGTGATGATGATGATGATGGCCGCCGCCTAGAATTTCAAGCACTTG | HindIII cut site |
| pBAD33*arsC^ec^* | GCTAGTCTAGACTGGCTGCGCTGGCGCTACGTC | XbaI cut site |
|  | GCTAGAAGCTTTTAGTGATGATGATGATGATGGCCGCCGCCTTTCAGGCGCTTACCCGCTTC | HindIII cut site |
| pBAD33/pBAD18/pHL100*varGvarHarsJ* | GCTAGGAGCTCGTTTAAAGGAAAAGGAAGATG | SacI cut site |
|  | GCTAGTCTAGATTATTTCAAATTCACTTGTGACGT | XbaI cut site |
| pBAD33/pBAD18/pHL100*arsB^ec^* | GCTAGGAGCTCTGTTTTATCCGGGAGGCATTATG | SacI cut site |
|  | GCTAGTCTAGATTACAAAGTGAAAGAGAGACGTAGC | XbaI cut site |
| pCB192Np*arsR* | GCTAGAAGCTTTGGTCAGATCCGGAAGGAAGC | HindIII cut site |
|  | GCTAGGAATTCCCTTTCATAATCAAACTCCAATATAT | EcoRI cut site |
| pCB192Np*varG* | \| ACCGAAGCTTTGAATGAGAGTC \| \| --- \| \|  \| | HindIII cut site |
|  | GCTAGGAATTCCCTTTCATCTTCCTTTTCCTTTAAAC | EcoRI cut site |
| pCB192Np*varH* | GCTAGAAGCTTACTGGGTATCGCACAAGCCTC | HindIII cut site |
|  | GCTAGGAATTCCCTTTCATTTTTTACTACTCCTTATG | EcoRI cut site |
| pCB192Np*arsJ* | GCTAGAAGCTTCATCCAACTTGGGAATTACCG | HindIII cut site |
|  | GCTAGGAATTCCCTTTCATAAACACTCTCTTAAAAAGAGC | EcoRI cut site |
| Pet28b*varG* | CGCCATATGATGGCAATTAAAGTCGGAATTA | NdeI cut site |
|  | TCGGAGCTCGATGCCAAGCCGACTTTACGA | EcoRI cut site |
| Pet28b*varH* | CGCCATATGACGCATCCAACTTGGGAATTAC | NdeI cut site |
|  | TCGGAATTCGGTTTGACGATTTGATGCACG | EcoRI cut site |
| Pet28b*varHC113G-R119G* | CGCCATATGACGCATCCAACTTGGGAATTAC | NdeI cut site |
|  | GTAAGCCAGT*TCC*ACCCGAACCTCCCAT*TCC*ATGCAAAGCGAC | OP primers |
|  | GTCGCTTTGCAT*GGA*ATGGGAGGTTCGGGT*GGA*ACTGGCTTAC | OP primers |
|  | TCGGAATTCGGTTTGACGATTTGATGCACG | EcoRI cut site |
| Pet28b*Gap* | CGCCATATGACTATCAAAGTAGGTATTAAC | NdeI cut site |
|  | TCGGAGCTCGACTTAGAGATGTGAGCGATC | EcoRI cut site |
| Pet28b*vc1041* | CGCCATATGCAGAAGGTACTCGTGGTGTGC | NdeI cut site |
|  | TCGGAATTCAGACGCTGGCCTTGCTGTTTTAG | EcoRI cut site |

***Restriction sites or linkers for overlapping PCR (OP) underlined and bold, respectively.
